# Mutations in the Second Alternative Oxidase Gene: A New Approach to Group *Aspergillus niger* Strains

**DOI:** 10.3390/jof9050570

**Published:** 2023-05-13

**Authors:** Michel Flipphi, Alexandra Márton, Vivien Bíró, Norbert Ág, Erzsébet Sándor, Erzsébet Fekete, Levente Karaffa

**Affiliations:** 1 Department of Biochemical Engineering, Faculty of Science and Technology, University of Debrecen, H-4032 Debrecen, Hungary(E.F.);; 2 Juhász-Nagy Pál Doctoral School of Biology and Environmental Sciences, University of Debrecen, H-4032 Debrecen, Hungary; 3 Institute of Food Science, Faculty of Agricultural and Food Science and Environmental Management, University of Debrecen, H-4032 Debrecen, Hungary

**Keywords:** ubiquinol:oxygen oxidoreductase encoding gene, *Aspergillus niger sensu stricto* complex, alleles, chromosomal deletion, retrotransposon insertion, point mutation, frameshift mutation, nonsense mutation, molecular taxonomy

## Abstract

Alternative oxidase is a terminal oxidase in the branched mitochondrial electron transport chain of most fungi including *Aspergillus niger* (subgenus Circumdati, section Nigri). A second, paralogous *aox* gene (*aoxB*) is extant in some *A. niger* isolates but also present in two divergent species of the subgenus Nidulantes—*A. calidoustus* and *A. implicatus*—as well as in *Penicillium swiecickii*. Black aspergilli are cosmopolitan opportunistic fungi that can cause diverse mycoses and acute aspergillosis in immunocompromised individuals. Amongst the approximately 75 genome-sequenced *A. niger* strains, *aoxB* features considerable sequence variation. Five mutations were identified that rationally affect transcription or function or terminally modify the gene product. One mutant allele that occurs in CBS 513.88 and *A. niger* neotype strain CBS 554.65 involves a chromosomal deletion that removes exon 1 and intron 1 from *aoxB*. Another *aoxB* allele results from retrotransposon integration. Three other alleles result from point mutations: a missense mutation of the start codon, a frameshift, and a nonsense mutation. *A. niger* strain ATCC 1015 has a full-length *aoxB* gene. The *A. niger sensu stricto* complex can thus be subdivided into six taxa according to extant *aoxB* allele, which may facilitate rapid and accurate identification of individual species

## 1. Introduction

For over a century, the filamentous Ascomycete fungus *Aspergillus niger* (subgenus Circumdati, section Nigri, series Nigri), a ubiquitous soil-borne saprophyte, has played a pivotal role in industrial biotechnology. Besides citric acid, *A. niger* also produces compounds such as gluconate [1], oxalate [2] and malate [3] and various native or heterologous enzymes [4] for diverse industrial applications. In addition to the native or heterologous production of single proteins, *A. niger* has become a platform for the synthesis of enzyme mixtures such as cocktails used for the degradation of plant polysaccharides [5]. The prominence of *A. niger* as a platform organism is not independent of the fact that it is classified as “generally regarded as safe” (GRAS) in the USA. However, black aspergilli, like most species of the extensive genus *Aspergillus*, are opportunistic fungi capable of colonizing the breathing tracts, trachea, nose and lungs of higher animals and humans, and can cause severe non-invasive mycoses and invasive aspergillosis in immunocompromised individuals and people suffering from respiratory maladies. Black aspergilli rank third among the causants of invasive aspergillosis in immunocompromised individuals; the (secondary) fungal infection complicates severe cases of COVID-19 in hospital treatment regimes. These fungi are notoriously difficult to separate by classical means, while the usual molecular taxonomy markers do not aid in reliable identification within the series Nigri of the section Nigri. Black aspergilli are cosmopolitan fungi, and clinical incidence of the respiratory disease symptoms is rising (e.g., [6,7,8]).

Joint molecular phylogeny of the calmodulin and *beta*-tubulin loci improved the resolution to a level which allowed for distinction with increased confidence to be made between *Aspergillus welwitschiae* and *A. niger* (*sensu stricto*), although some isolates could not be unambiguously assigned to the one or the other of these sibling taxa [8,9,10]. However, this resolution was clearly insufficient to identify diverse clades within the *A. niger sensu stricto* complex, although rapid and accurate identification of individual species could be crucial in clinical settings.

The alternative terminal oxidase (systematic name: ubiquinol:oxygen oxidoreductase, non-electrogenic; EC 1.10.3.11) is encoded in the nuclear genome but located in the mitochondrion, anchored in the inner mitochondrial membrane (e.g., [11,12]). It provides an alternative for the cytochromic electron flow and bypasses the proton-pumping complexes III and IV of the canonical electron transfer chain [13] (Supplementary Figure S1). While alternative oxidase (Aox) has been associated with various apparently nonessential physiological functions, the consequence of the direct transfer of electrons from ubiquinol to oxygen without concomitant proton translocation is the uncoupling of ATP synthesis via oxidative phosporylation from NADH reoxidation, to allow carbon catabolism to continue unabated when ATP demand is low (e.g., during slow growth: [14]). For this reason it plays an important role in the energetics of overflow metabolisms: alternative oxidase activity is positively correlated with the final yield of industrial bioprocesses as *A. niger* citric acid [15,16], *Aspergillus terreus* itaconic acid [17] or *Acremonium chrysogenum* cephalosporin-C fermentation [18], but also with the detrimental production of polyketide mycotoxins (e.g., sterigmatocystin, aflatoxin) by, e.g., *Aspergillus nidulans* [19] and *Aspergillus flavus* [12]. Understanding the origin, structure and evolution of alternative respiratory pathways and their key non-electrogenic enzymes could therefore contribute to strain improvement efforts in industrial biotechnology.

Alternative oxidase (Aox) is near ubiquitous in the fungal kingdom, although it is not an essential enzyme activity under all growth conditions and lifestyles. For instance, the so-called genetic model yeasts *Saccharomyces cerevisiae* and *Schizosaccharomyces pombe* do not have Aox. Conversely, the co-existence of paralogue alternative oxidase genes is rarely described in fungi. Two neighbouring genes, *AOX1a* and*AOX1b*, are orientated in tandem in the ascomycete yeast *Candida albicans* [20,21]. On the other hand, the filamentous model fungus *Neurospora crassa* has two sequence-related but genetically unlinked alternative oxidase genes, *aod-1* and *aod-3* [22,23].

In *Aspergillus niger* strain WU-2223L (recently reappreciated as an isolate of *Aspergillus tubingensis*), a full-length alternative oxidase cDNA was reported [24,25] (accession number of the cDNA: AB016540). The identified *aoxA* gene encodes a protein of 351 amino acids (AA) (BAA32033 and BAB03469) with a predicted N-terminal mitochondrial target sequence of 53 amino acids. *aoxA* has two phase-two introns bounding an exon of 291 base pairs (bp). This gene model is conserved in all Eurotiales and Onygenales for which genome sequences are available (in June 2022; results not shown). With the publication of the first two *Aspergillus niger* whole genome sequences in 2007 and 2011 [26,27], it became evident that there are also paralogous *aox* sequences in some strains of *Aspergillus niger* that were apparently absent from other *Aspergillus niger* isolates. The current availability of dozens of whole genome sequences of *Aspergillus niger* strains and other species in the Nigri series of the section Nigri (for molecular taxonomic relations within the Aspergillaceae, see [28]) allows for investigation of the presence, the status and the origin of the second alternative oxidase gene in the *A. niger* complex (*A. niger sensu stricto*: cf. [10,29]). In the current work, we show that the paralogous *aoxB* gene in some 75 genome-sequenced *A. niger* strains features variations which rationally affect the transcription or the function of *aoxB*, or at the least, alter the encoded protein at one of its termini. Based on five *aoxB* mutant alleles, one could subdivide the *A. niger sensu stricto* complex into six taxa. Moreover, the presence of an *aoxB* pseudogene distinguishes *A. tubingensis* strains (*A. niger sensu lato*) from other named species in the *A. luchuensis* clade. *aoxB* could, thus, be used as a new diagnostic marker unique to a narrow taxon of black aspergilli, which allows for distinction between multiple clades within the *A. niger sensu stricto* complex in clinical and industrial settings. To date, confident separation could only be accomplished after comparative analyses of whole genome sequences. 

## 2. Materials and Methods

### 2.1. Mining of Alternative Oxidase and Type-II NADH:Ubiquinone Oxidoreductase Genes, Gene Synteny around Gene Loci and Confirmation of Expression

The coding sequences of alternative oxidase and type-II NADH:ubiquinone oxidoreductase genes (ATG–STOP) were mined following TBLASTN screening of DNA databases on the National Center for Biotechnology Information (NCBI) servers (primarily the Whole Genome Shotgun contigs (WGS) database, which includes the genomes of approximately 75 different *A. niger* strains and isolates) using the available online tools [30]. The TBLASTN query sequences are specified in the Results and Discussion section of this paper. Using the AoxA protein from WU-2223L [24,25] as the query, a second alternative oxidase gene was searched for, apparently encoding peptides with 55–65% amino acid identity to the query protein (in particular, in exons 2 and 3). The N-terminal mitochondrial signal and membrane anchor in exon 1 are far less sequence-conserved between Aox paralogues coexisting in the same fungus. TBLASTN screens were run with near-default settings, although the Expect threshold stringency was lowered to 1000 and the Gap Cost reduced to Existence 10 and Extension 1, while composition adjustment was omitted, and low complexity regions were not filtered. For a few fungi (including *A. niger* CBS 513.88), the genome sequences are located in the Refseq genome database, and these were screened using the BLAST Genomes module. We did not use the results of automated annotation at NCBI (“Models” or “mRNA” at nr/nt), nor did we mine deduced protein databases for computer-annotated sequences.

We included species exclusively available from the MycoCosm depository of fungal genome sequences of the U.S. Dept. of Energy Joint Genome Institute (JGI) (https://mycocosm.jgi.doe.gov/mycocosm/home) (accessed on 5 November 2022) [31]. For the JGI screens, search criteria were narrowed and second alternative oxidase genes—having 50–75% identity at the amino acid level with *A. niger* ATCC 1015 AoxB—were searched for that neighbour divergently orientatedparalogues (AndB) of type-II alternative NADH dehydrogenase genes, which (themselves) are 45–55% (amino acid level) identical to the ubiquitous alternative NADH dehydrogenase (AndA), present in all genome-sequenced species of the Eurotiomycetidae subclass. For two species of Aspergillaceae, we obtained permission to use their JGI-lodged genome assemblies to identify the paralogous *aox* and *and* genes and to deduce their gene models (Supplementary Table S1) guided by the conservation of intron positions observed in these paralogues in Eurotiomycetidae taxa. These two species were *Aspergillus implicatus* strain CBS 484.95 (project ID: 1052426) and *Penicillium swiecickii* strain 182_6C1 (project ID: 1144761). We confirmed the absence of the *aoxB*–*andB* gene couple from a group of species closely related to *Aspergillus calidoustus* (same series) and exclusively available at the JGI genome sequence depository: *A. carlsbadensis* CBS 123894, *A. germanicus* CBS 123887, *A. insuetus* CBS 107.25, *A. keveii* CBS 209.92 and *A. pseudodeflectus* CBS 756.74. 

To corroborate orthology amongst homologous genes, TBLASTN hits and their local environment were inspected for intron positional conservation (cf. [32]) and for colinearity with neighbouring genes that could imply gene clustering. The genome browsers of the respective JGI genomes were used to inspect the direct environments of the *aoxB* locus for gene synteny in *Aspergillus niger* ATCC 1015 (https://mycocosm.jgi.doe.gov/Aspni7/Aspni7.home.html), *A. calidoustus* SF006504 (https://mycocosm.jgi.doe.gov/Aspcal1/Aspcal1.home.html), *A. implicatus* CBS 484.95 (https://mycocosm.jgi.doe.gov/Aspimp1/Aspimp1.home.html) and *P. swiecickii* 182_6C1 (https://mycocosm.jgi.doe.gov/Penswi1/Penswi1.home.html). We identified perfectly matching *A. calidoustus*, *A. implicatus* and *A. niger* RNA sequence reads (SRAs) confirming intron excision (listed in Supplementary Table S2) by BLASTN screening the species-designated Sequence Read Archives at NCBI, using 60 nt long query sequences covering the exon fusion site produced by the predicted intron excision. To generate estimations of sequence similarity (% of amino acid or DNA identity) we ran Clustal Omega multiple sequence alignments [33] that produce percent identity matrices as part of the output.

### 2.2. Maximum-Likelihood Phylogenetic Analysis

Different selections of Eurotiomycetidae and Lecanoromycetes Aox proteins were first aligned using multiple sequence alignment with fast Fourier transform (MAFFT, version 7) [34,35] using E-INS-i iterative refinement, trained to recognize multiple conserved domains with large spacing, and BLOSUM45 as the scoring matrix (fixed variables). The resulting alternative alignments were subsequently trimmed using BMGE (block mapping and gathering using entropy: [36]) to optimize the ensemble of the highly informative regions while deleting less similar areas harbouring many of the gaps introduced during alignment, utilizing the amino acid substitution matrix BLOSUM55 and a block size of 4 (fixed settings). BMGE-trimmed alignments were subsequently used to construct maximum-likelihood (ML) trees with PhyML (version 3) (online module) [37], initially employing the general amino acid replacement matrix LG [38] with the following settings: invariable sites, estimated; substitution rate, gamma; number of substitutions, 4; gamma-shape, estimated. However, using the Aikake information criterion (AIC), the updated automated Smart Model Selection module of PhyML [39] currently selects the kingdom-specific amino acid replacement matrix, Q.plant [40], as the recommended model. We addressed the apparent inconsistency of using a “plant-specific” amino acid substitution model by estimating the evolutionary relationships between filamentous fungal AoxA and AoxB paralogues from the same trimmed alignment in parallel with either the LG or the Q.plant matrices. Maximum-likelihood estimations were used consistently in an effort to make our analyses compatible with published molecular taxonomy studies of black aspergilli (for example, [8,9,10]).

ML trees were drawn with FigTree version 1.4.3 and rooted in the designated outgroup of Lecanoromycetes AoxA; the outgroup was subsequently eliminated from view by subtree selection in the tree-drawing program. For the convenience of the reader, the selected maximum-likelihood tree shows the stable placement of the paralogous AoxB clade across the alternative phylogenies along the collapsed monophyletic AoxA clades for species belonging to each of the Eurotiales families Aspergillaceae, Thermoascaceae, Elaphomycetaceae, or Trichocomaceae, or species belonging to the Onygenales order. Branch stability was assessed with approximate Likelihood Ratio Tests (aLRTs) [41] integral to PhyML operation using the default settings. 

### 2.3. Aspergillus niger Strains Used for Sequence Verification of aoxB Alleles Reported in this Study

The strains used to identify the six *aoxB* alleles are listed in Table 1, along with their original source and the accession numbers of their sequences. The sequences we determined and submitted to GenBank are from ATG to STOP for the *aoxB* alleles, except for allele II from which the 5′-quarter (23%) of the *aoxB* coding region has been removed by the chromosomal deletion of 2294 nt typical for allele II (see Results and Discussion section of this paper). cDNA proves excision of two introns from the *aoxB* pre-mRNA (ATCC 1015) which are position-conserved with those in the *aoxA* pre-mRNA in WU-2223L. 

### 2.4. Isolation of A. niger Genomic DNA; Polymerase Chain Reaction (PCR), Vector Cloning and Sequence Determination

Different *A. niger* strains (Table 1) were grown on minimal medium (2.50 g (NH_4_)_2_SO_4_; 0.15 g KH_2_PO_4_; 0.15 g NaCl; 2.25 g MgSO_4_·7H_2_O; 1.50 mg Zn^2+^; 10 g D-glucose; 0.10 mg Fe^2+^; 0.06 mg Cu^2+^ and 0.05 mg Mn^2+^, per litre) for 24 h in 500 mL Erlenmeyer flasks (VWR International Kft., Debrecen, Hungary) containing 100 mL of liquid in a rotary shaker (Infors AG, Basel, Switzerland) at 250 revolutions per minute (rpm) at 30 °C. Cultures were inoculated with high density conidiospore suspensions in a 0.01% Tween-20 solution. Mycelia were harvested from three independent cultures (three independent biological replicates) by filtration over Miracloth (Millipore, Merck KGaA, Darmstadt, Germany), washed with distilled water and deep frozen in liquid nitrogen. Genomic DNA (gDNA) was isolated using the Macherey-Nagel NucleoSpin Plant II kit (Macherey-Nagel GmbH & Co., KG, Düren, Germany).

Targeted PCR reactions using a gDNA template were performed with gene-specific oligonucleotide primer pairs (Supplementary Table S3) (Integrated DNA Technologies, Leuven, Belgium) and DreamTaq DNA Polymerase (Thermo Scientific, Thermo Fisher Scientific, Waltham, MA, USA) in a T100TM Thermal Cycler (Bio-Rad, Bio-Rad Hungary Ltd., Budapest, Hungary). Cycling conditions after initial denaturation at 95 °C for 3 min: 35 cycles of 95 °C for 30 s, 54 °C for 1 min and 72 °C for 0.5–1 min, followed by post-cyclic elongation at 72 °C for 5 min. Purified PCR fragments (NucleoSpin Gel & PCR Clean-up, Macherey-Nagel) were cloned into pGEM-T Easy (pGEM-T Easy Vector System I, Promega Corporation, Madison, WI, USA). Plasmid DNA was isolated using the NucleoSpin Plasmid EasyPure kit (Macherey-Nagel). DNA from three independent clones (three technical replicates) was sequenced over both strands using universal primers hybridizing to the vector (Eurofins Genomics, Ebersberg, Germany). The determined *aoxB* and *andB* sequences were deposited at GenBank (see Table 1 for the respective accession numbers).

### 2.5. Isolation of A. niger Total RNA for cDNA Sequence Analysis

Total RNA for first-strand cDNA synthesis was isolated from cultures of ATCC 1015. The biomass was grown under manganese paucity in 500 mL Erlenmeyer flasks (VWR International Kft., Debrecen, Hungary) with 100 mL of growth medium (2.50 g (NH_4_)_2_SO_4_; 0.15 g KH_2_PO_4_; 0.15 g NaCl; 2.25 g MgSO_4_·7H_2_O; 1.50 mg Zn^2+^; 140 g D-glucose; 0.10 mg Fe^2+^ and 0.06 mg Cu^2+^, per litre) (i.e., no MnCl_2_ added) in a rotary shaker (Infors AG, Basel, Switzerland) at 250 rpm at 30 °C. Total RNA was isolated using the RNA Plant kit (Macherey-Nagel GmbH & Co., KG, Düren, Germany). First-strand cDNA was synthesized from a total RNA template with Oligo(dT) as the primer using the RevertAid First Strand cDNA Synthesis Kit (Thermo Scientific, Thermo Fisher Scientific, Waltham, MA, USA). First strand cDNA was subsequently used as the template for the PCR reaction, and the cycling conditions after initial denaturation at 95 °C (3 min) were as follows: 35 cycles of 95 °C for 30 s, 54 °C for 1 min, and 72 °C for 0.5–1 min, followed by one post-cyclic elongation at 72 °C (5 min). The downstream sequencing procedure after cloning of the RT-PCR amplification was the same as described for gDNA (above). All RT-PCR experiments were performed in triplicate, starting with biomass from three independent liquid cultures.

## 3. Results and Discussion

### 3.1. A Rare Alternative Oxidase Paralogous Gene (aoxB) Occurs in the Aspergillus niger Species Complex (Sensu Stricto) as well as in Distally Related Aspergillus calidoustus

Comparative analysis allowed for resolution of the gene model of a full-length gene (*aoxB*) for a paralogue alternative oxidase of 347 amino acids in *A. niger* strain ATCC 1015 as well as in the sibling species *Aspergillus welwitschiae* (strain CCMB 674) and *Aspergillus awamori* (strain IFM 58123). The predicted gene models of the three *aoxB* genes are detailed in Supplementary Table S1. A schematic overview of the *aoxB* locus in ATCC 1015 is shown in Supplementary Figure S2, including its intron–exon structure and that of the divergently orientated neighbouring gene. The *aoxB* gene in these three genomes has two phase-two introns, strictly position-conserved with those in the ubiquitous *aoxA* gene in the same fungi, bounding the central exon of 291 bp in both *aoxA* and *aoxB* (Supplementary Figure S2a). Moreover, the coding part of the 3′ exon is 511 bp in both paralogous genes. The two-intron *aoxB* gene model in ATCC 1015 was confirmed with overlapping cDNA clones obtained after RT-PCR-amplification (GenBank accession OQ686795). The *aoxB* DNA sequences (ATG–STOP) are 100% identical in ATCC 1015, NRRL3 (also known as N400) and N402, 98% identical to those in IFM 58123 and 96 % identical to those in CCMB 674. The paralogous AoxB protein in *A. niger* ATCC 1015 (347 residues) is >57% identical to the ubiquitous AoxA (351 residues) in the same strain. The similarity increases to >63% when the predicted N-terminal mitochondrial transfer peptide (~50 AA) is removed from both proteins.

A very similar *aoxB* paralogue (1159 bp start-to-stop, including introns) is found in all six accessible genomes of *Aspergillus calidoustus* (strain SF006504 [42]: WGS Master Accession CDMC), a species of series Calidousti, section Usti, in the subgenus Nidulantes (rather than Circumdati, the subgenus to which *A. niger* belongs). The two phase-two introns are position-conserved; exon 2 and the coding part of exon 3 are exactly the same size as in *A. niger* ATCC 1015, at 291 and 511 bp, respectively. The overall similarity between *aoxB* in ATCC 1015 and in *A. calidoustus* at the DNA level (including introns) is ~70% identical. The *A. calidoustus* AoxB protein is predicted to be 345 AA long and is almost 74% identical to the orthologous *A. niger* ATCC 1015 AoxB over the complete sequence. We did not encounter full-length *aoxB* orthologues in other Black Aspergilli (for which the genome sequence has been published), all of which do harbour the ubiquitous *aoxA* gene. Moreover, we did not find an *aoxB* orthologue in the other species of section Usti available at NCBI, neither in *Aspergillus ustus* nor in *Aspergillus* sp. ADI-1, nor in five species of the series Calidousti for which genome sequences have been lodged at the JGI, namely *A. carlsbadensis*, *A. germanicus*, *A. insuetus*, *A. keveii* and *A. pseudodeflectus*.

### 3.2. aoxB Is Divergently Orientated from a Rare Paralogous Gene Encoding a Type-II Alternative NADH:Ubiquinone Reductase

**Figure 1:**
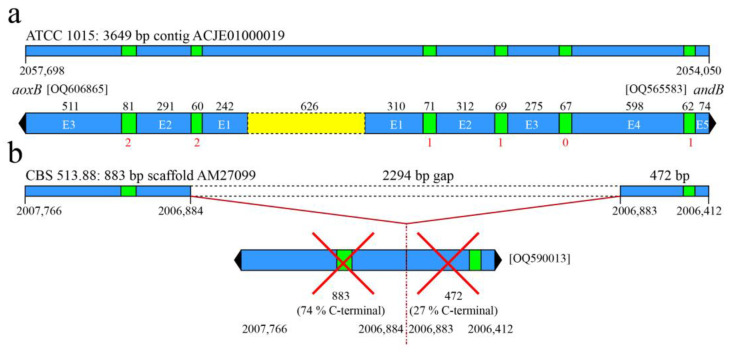
Schematic views of the divergently orientated *aoxB* and *andB* genes in *Aspergillus niger* ATCC 1015 and the chromosomal deletion eliminating the gene function of both these genes in *Aspergillus niger* CBS 513.88. (**a**) The intact *aoxB*–*andB* gene couple in ATCC 1015. The 626 bp sequence between the opposite start codons of the coupled genes is highlighted in yellow. The exact location of the gene couple on sequence contig ACJE01000019 is given. (**b**) The consequences of a deletion of a 2294 bp sequence in *Aspergillus niger* strain CBS 513.88 corresponding to *aoxB* allele II. The deletion affects the *aoxB* and *andB* genes at their respective 5′ ends and eliminates the intergenic region. Conceivably, neither of the 5′ truncated genes are expressed. The corresponding genome sequence in CBS 513.88 is shown by the interrupted bar, labelled with the four coordinates defining the remaining parts of deletion-interrupted loci on sequence scaffold AM27099. Relevant GenBank accession numbers certifying the local genome sequences are given.

When we inspected the *direct* environment of the locus of the rare *aoxB* orthologue in *A. niger* ATCC 1015 and *A. calidoustus*, it was found that *aoxB* is divergently orientated from a gene encoding an equally rare paralogue of a type-II NADH dehydrogenase or alternative NADH:ubiquinone reductase (non-electrogenic). Figure 1 schematically shows the divergently orientated genes in *A. niger* ATCC 1015. In Supplementary Figure S2, the proposed intron–exon structure (4 introns) of the divergently orientated gene has been depicted for *A. niger* ATCC 1015 opposite that of *aoxB*, and in Supplementary Table S1 the coordinates of the exons are provided. The *andB* intron–exon structure was confirmed by comparative analysis and by cDNA sequencing (GenBank OQ689783). This divergent gene couple appears to be unique in the NCBI databases for the two different narrow *Aspergillus* taxa mentioned above.

Type-II NADH dehydrogenase (EC 1.6.5.9) re-oxidizes NADH to NAD^+^ but transfers the electrons to ubiquinone without pumping protons out of the mitochondrion (see [43] for a recent review). The rare alternative NADH dehydrogenase paralogue in the divergent gene couple extant in {*A. niger* ATCC 1015–*A. welwitschiae*–*A. awamori*} and in *A. calidoustus* we have named *andB*, and the unlinked ubiquitous gene for the enzyme activity present in all species of Eurotiomycetidae and Lecanoromycetes we have named *andA*. The *A. niger* ubiquitous *andA* gene is the structural orthologue of the *A. nidulans* gene at locus AN1094 (~85% AA identity) and the *N. crassa* gene at locus NCU08980 (~65% AA identity). The *A. nidulans* and *N. crassa* orthologous enzymes oxidize cytosolic NADH, i.e., they are non-proton-pumping type-II NADH dehydrogenases located on the cytosolic side of the mitochondrial inner membrane [44,45].

The two paralogue alternative NADH dehydrogenases (AndA and AndB) are ~51% identical in both *A. niger* ATCC 1015 and *A. calidoustus* (for the full-length gene products). The two orthologous AndB peptides are ~67% identical, much lower than >83% in the case of the AndA gene products. A stand-out difference between the paralogous *and* genes is the absence of the third (phase zero) intron from the ubiquitous “A” genes (cf. Supplementary Figure S2b).

### 3.3. The aoxB and andB Gene Couple Occurs Sporadically in Four Dispersed Taxa of Aspergillaceae

The rare occurrence of the coupled paralogous *aoxB* and *andB* genes was further investigated by searching the MycoCosm genome sequence depository at the Joint Genome Institute (U.S. Department of Energy) [31]. Amongst the ~300 species of *Aspergillus* and *Penicillium* deposited, we found two additional species in which the rare gene couple *aoxB*–*andB* was present. *Aspergillus implicatus* belongs to the section Sparsi, series Implicati, an early divergent taxon of the subgenus Nidulantes. On the other hand, *Penicillium swiecickii* is a species of the Penicillium subgenus section Ramosum, series Lanosa, long separated from the three *Aspergillus* species that also harbour the rare gene couple. The gene models of the coupled genes we predict in these two Aspergillaceae are detailed in Supplementary Table S1, giving the JGI scaffold numbers on which they occur and the coordinates of the exons in between the startcodon and the stopcodon. See [28] for the assignment of these four species to *Aspergillus*/*Penicillium* subgenera, sections and series. 

**Figure 2:**
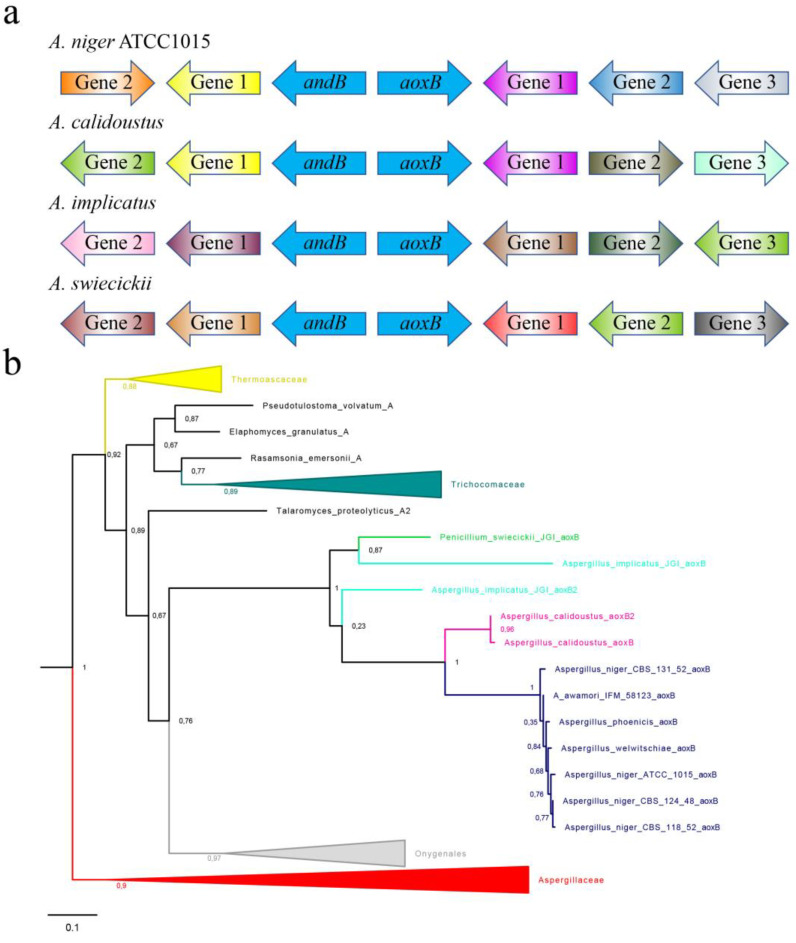
Origin of the rare *aoxB*–*andB* divergent gene couple present in four scattered taxa of Aspergillaceae. (**a**) Gene synteny near the *aoxB*–*andB* locus in *A. niger* ATCC 1015, *A. calidoustus*, *A. implicatus* and *P. swiecickii*. The three auto-annotated loci (filtered models) on each side of *aoxB* were taken from the respective genome browsers on the JGI website (see Materials and Methods section for details of the appropriate websites). The orientation of the neighbouring genes was indicated by the arrow, but for the sake of simplicity, the absolute size of the predicted coding regions, the intergenic distances and the deduced intron–exon structures were ignored in the schematic summary shown here. The predicted functions of the neighbouring genes were colour-coded such that genes with identical annotated function could easily be discerned. The gene at the left of *aoxB* is the divergently orientated alternative NADH dehydrogenase *andB.* In *A. niger* ATCC 1015 and *A. calidoustus*, the two genes directly neighbouring the *aoxB*–*andB* gene couple (divergent blue arrows) are also orthologous, encoding a zinc-cluster transcriptional regulator (~700 AA) next to *aoxB*, and a ~600 AA-sized protein with an N-terminal 3-hydroxyacyl-CoA dehydrogenase domain downstream of *andB*. The synteny suggests the existence a four-membered gene cluster in ATCC 1015 and *A. calidoustus*. (**b**) Estimation of the origin of the AoxB-encoding sequences in the order of the Eurotiales. A maximum-likelihood phylogenetic analysis was performed after multiple sequence alignment (MAFFT) of 344 ubiquitous alternative oxidase proteins (i.e., all orthologous AoxA) supplemented with 12 paralogous AoxB proteins from the *Aspergillus niger sensu strictu* complex, its sibling species *A. awamori* and *A. welwitschiae*, two sequence variants of *A. calidoustus*, *A. implicatus*, and *P. swiecickii*. All these fungi have one *aoxB* gene except *A. implicatus* (cf. Supplementary Table S1). The alignment was trimmed using BMGE to 290 informative residues and subsequently used to infer maximum-likelihood trees using PhyML employing the LG substitution model. Stability of branches was assessed with approximate Likelihood Ratio Tests. See the Materials and Methods section for experimental details and references. For clarity, only the sector of the tree in which the AoxB-specific clade clusters with well-defined AoxA clades is shown. The clades specific to Aspergillaceae, Thermoascaceae, Elaphomycetaceae, Trichocomaceae (all families of Eurotiales), and Onygenales were collapsed for a more convenient overview.

Further inspection of the direct environment of the coupled *aoxB*–*andB* loci (Figure 2a) suggests that the two genes directly neighbouring the divergent couple could be part of a four-gene cluster found in {*A. niger* ATCC 1015–*A. welwitschiae*–*A. awamori*} as well as *A. calidoustus*. The seven-exon gene of a putative zinc-cluster transcriptional factor is located downstream of *aoxB* and transcribed from the opposite strand (pink gene labelled “gene-1” in Figure 2a): the zinc-cluster regulator proteins are 60% identical over their complete width. Downstream of *andB* and on the same strand is a two-exon gene for a protein which is ~ 600 amino acids long with an N-terminal 3-hydroxyacyl-CoA dehydrogenase domain (yellow gene labelled “gene-1” in Figure 2a). The orthologues in ATCC 1015 and *A. calidoustus* are >80% identical. 

To test whether the coupled *aoxB*–*andB* genes are co-expressed and thus potentially part of a functional gene cluster, we screened extant sequence read archives available at NCBI (generated by third parties but freely accessible). We screened SRA libraries (RNA reads) in *A. calidoustus* and *A. implicatus* for sequence reads that cover the predicted exon–exon fusions after intron excision of pre-mRNAs for *aoxB* and *andB*. We also identified SRA reads that confirm intron excision for both genes in a set of *A. niger* gene deletion strains, generated in the NRRL3 background which is in the same pedigree as ATCC 1015 (i.e., wild type for *aoxB*). Supplementary Table S2 lists individual reads which cover exon–exon fusions. We found pre-mRNA splicing for all six predicted introns in *A. calidoustus* as well as in *A. niger* (strains *delta-araR*, *delta-gaaR*, *delta-galX*, *delta-rhaR*, *delta-xlnR* in the NRRL3 background) grown on complex carbon sources. In *A. implicatus*, we found SRA reads that cover the excision of either of the *aoxB* introns and reads that cover the exon–exon fusions of introns 2 and 4 of the *andB* gene. Note that the expression rate is highly dependent of the growth conditions under which these sequence read resources were created, and therefore quantitative analyses of covering SRAs are not informative.

### 3.4. The Rare aoxB Paralogue Appears to Originate from an Unidentified Host Taxon within the Eurotiomycetidae but Not an Aspergillaceae Taxon

To investigate the origins of the *aoxB* paralogue in the rare *aoxB*–*andB* gene couple, we collected the DNA coding for some 350 Aoxs from Eurotiomycetidae and Lecanoromycetes genomes, following TBLASTN screening of the NCBI’s Whole Genome Shotgun database in May 2022, using the ubiquitous *A. niger* ATCC 1015 AoxA protein as the query sequence. After manual deduction of the intron–exon structures and subsequent translation of the open reading frame after in silico intron removal, we generated alternative maximum-likelihood trees with variably sized AoxA content and 12 full-length AoxB paralogues (the latter specified in Figure 2b). Two different substitution models, Q.plant and LG, were applied in parallel in estimating multiple maximum-likelihood phylogenies from multiple sequence alignments of differently defined batches of Aox proteins using the PhyML (v3.0) online facilities (see Materials and Methods section for more details). The alternative trees were all rooted on the clade of Lecanoromycetes AoxA proteins as the default outgroup. The relevant section of one representative maximum-likelihood tree is shown in Figure 2b. Despite the scattered and sporadic occurrence, all alternative AoxA–AoxB phylogenies consistently predict essentially the same origin for *aoxB*, with the grouped AoxB proteins acting as a sister clade to the Onygenales AoxA proteins in a distinctive subtree further consistent with the AoxAs from species in the (other) Eurotiales families—Trichocomaceae, Elaphomycetaceae and Thermoascaceae. The genome-sequenced species in the aforementioned four taxa (i.e., those outside Aspergillaceae) do not feature *aox* paralogues, with the exception of *Rasamsonia emersonii*, whose paralogue is not intimately related with AoxB. In *A. implicatus*, a secondary duplication is likely at the origin of the two paralogue Aoxs that evolved within the diminutive but well-defined AoxB clade. In Figure 2b, the third paralogue not linked to any alternative NADH dehydrogenase gene is called AoxB2. This third *aox* paralogous gene in *A. implicatus* is also expressed (Supplementary Table S2).

The underlying topology of the ubiquitous AoxA was revealed by inferring maximum-likelihood trees from orthologous “A” proteins only (i.e., without AoxB) using exactly the same settings for alignment, trimming and tree inference as for the mixed paralogue phylogenies. Supplementary Figure S3 shows that the taxonomically aberrant position of the Onygenales AoxA clade, clustering amongst the Trichocomaceae, Elaphomycetaceae and Thermoascaceae family clades of the Eurotiales instead of forming a discernible outgroup to all Eurotiales, is not due to the presence of the AoxBs in the mixed phylogenies.

One characteristic differentiating the ubiquitous *andA* gene from its *andB* paralogue is the intron–exon structure (cf. Supplementary Figure S2b). In *A. niger* ATCC 1015, *A. calidoustus* and *A. implicatus*, *andB* has four introns at strictly conserved gene positions; *P. swiecickii* lacks an intron at the first of those positions. By contrast, the ubiquitous *andA* gene lacks the phase-zero intron at the third conserved position in all four species harbouring the *aoxB*–*andB* gene couple. In fact, the third intron position (phase zero) is not occupied in the *andA* orthologous gene in all genome-sequenced species of *Penicillium* as well as in the overwhelming majority of genome-sequenced *Aspergillus* species: the exceptions are *A. wentii* (section Cremei), *Aspergillus* sp. HF37 and *A. sclerotiales* (section Polypaecilum). In all other taxa of Eurotiomycetidae, including the remaining Aspergillaceae taxa (i.e., *Penicilliopsis*, *Evansstolkia*, *Monascus*, *Xeromyces* and “*Byssochlamys*” sp. BYSS01) as well as all the sequenced Thermoascaceae, Trichocomaceae, Elaphomycetaceae and Onygenales, the ubiquitous *andA* gene also has all four intron positions occupied. The change in the gene model of the *andA* gene—the loss of the phase-zero intron at conserved position 3 in all Penicillia and in almost all Aspergilli—provides circumstantial evidence that the origin of the present day *aoxB*–*andB* couple is amongst the divergent families of Eurotiales beyond the divergence of the Aspergillaceae. 

### 3.5. Multiple Alleles for the Acquired aoxB Paralogue in the Aspergillus niger Sensu Stricto Complex

**Figure 3:**
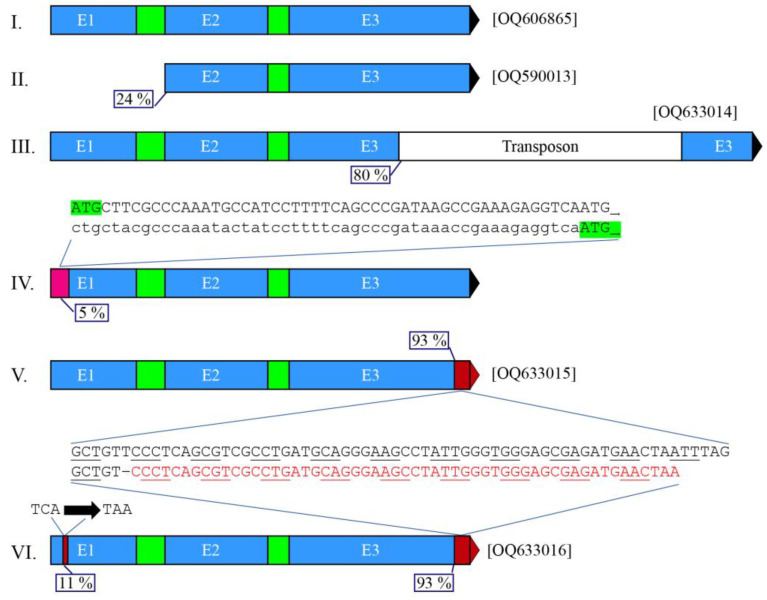
Schematic overview of the six alleles of the *aoxB* paralogous gene in the *Aspergillus niger sensu stricto* complex for which whole genome sequences were accessible at the NCBI’s Whole Genome Shotgun (WGS) database (in September 2022). The six alleles (5′ to 3′) are indicated by the roman numbers I–VI. The full-length wild-type *aoxB* allele (Allele I) is present in the strains listed under Group I in Table 2 and include strain ATCC 1015 (GenBank OQ606865). The positions of the mutation or interruption in the *aoxB* coding region are indicated by the boxed % of CDS. Alleles IV, V and VI are the consequences of point mutations shown in the three sequence inlets. For Allele IV, the use of a downstream start codon (relevant ATGs in green) is the predictable consequence of the observed missense mutation (A–C transversion) that eliminates the wild-type start codon, N-terminally truncating the mitochondrial target sequence (53 AA) by one third. For Allele V, the deletion of one T frameshifts the CDS near its 3′ end, indicated by alternate underlining of the used codons in the wild-type and the mutant alleles, the red sequence occurring after the frameshift in *aoxB* alleles V and VI. Allele VI is a double mutant allele derived from allele V, as it features an additional nonsense mutation (in codon number 38) near the 5′ of the *aoxB* coding region.

Interestingly, the acquired *aoxB* gene in genome-sequenced strains of *A. niger sensu stricto* features considerable genetic variation. Amongst the genome-sequenced strains, we found five different mutations that severely affect the encoded enzyme or, at the least, terminally modify the *aoxB* gene product. Figure 3 provides details about the six alleles and the mutations at their basis. Table 2 lists the *A. niger* strains that carry the six *aoxB* alleles identified. Group VI consists of two strains that have accumulated a nonsense mutation in the background of the pre-extant frameshift mutant allele V. All five other groups contain strains of opposite mating type (cf. [29,46]). We have confirmed by PCR amplification and sequencing that five of these *aoxB* alleles are indeed identical to those originally identified in silico in the corresponding genome sequences. In Table 1, the corresponding GenBank accession numbers certifying various *aoxB* alleles (including the wild type allele) are listed together with the strains taken as typical for each of these *aoxB* alleles. Nine *A. niger* strains listed in Table 2 in Group IV—sequenced by three different groups of scientists—carry the defining A–C transversion that would eliminate the original start codon. Their *aoxB* sequences (1185 bp) are identical except for one nucleotide deep within the first intron (i.e., position 280).

The wild-type *aoxB* allele potentially produces the full-length AoxB paralogue of 347 AA in length in, amongst others, *A. niger* strain ATCC 1015 [27] and in the laboratory strains NRRL 3 and N402. Full-length AoxB is marginally shorter at the N-terminus than the ubiquitous AoxA protein (351 AA), there where the mitochondrial target sequence is located. This full-length allele is also present in the sibling species *A. welwitschiae* [47] (5 strains) and *A. awamori* IFM 58123 [48], in addition to 14 genome-sequenced *Aspergillus niger sensu stricto* strains. The genome sequences of “*Penicillium fimorum*” strain S/N-308-OC-P1 (Master accession number JACVQR) are likely contaminated with the DNA of an *A. niger*-like fungus, specifying two non-identical *aoxA* genes and one full-length *aoxB* orthologue.

The most dramatic of the mutant *aoxB* alleles concerns a chromosomal deletion of 2294 bp that removed exon 1 and intron 1 (i.e., everything before codon Tyr82) (Figure 3, group II), but also the complete intergenic region (626 bp) as well as approximately 76% of the coding regions of the divergently orientated *andB* gene. This deletion thus eliminates expression of both genes. Figure 1 outlines the situation in CBS 513.88, the first *A. niger* strain that was genome-sequenced [26]. This severe dual-loss-of-function mutation is also present in the neotype strain CBS 554.65 [46] (verified: GenBank OQ590013), as well as in *A. lacticoffeatus* (CBS 101883) and *A. niger* Van Tieghem (ATCC 13496) [49]. The *aoxB* deletion (allele II) is present in 13 genome-sequenced strains.

The second *aoxB* mutation involves the integration of a non-autonomous copy of a retrotransposon in the third exon at ~80% of the coding sequences (Figure 3: Group III). The codons of amino acids R276 and M277 are included in the 7 nt long target-site duplication (TSD) 5′-GAGAATG generated by the integration of an ~13 kb long type-1 element. The *aoxB* transposon interruption occurs in 16 genome-sequenced strains. The retrotransposon features long terminal repeats (LTRs: direct repeats) of ~158–162 bp which are 72.84% identical. Supplementary Figure S4 shows the LTRs of the eight copies of this transposon (eight different TSDs) found in one or more of the 16 strains in which the *aoxB* gene is retrotransposon interrupted. In CBS 147324, an autonomous copy is present, 5127 nt long with 100% identical, 159 nt long LTRs between hexamer TSDs 5′-AGAAAC. This RNA transposon identifies as a member of the Ty1/copia family [50]. The autonomous copy contains the 4512 nt long open-reading frame encoding a multifunctional protein (1503 AA) with integrase, reverse transcriptase and RNase H domains in the order typical of the Ty1/copia family (Supplementary Figure S5). The Ty1/copia transposon in CBS 147324 has not been reported before: its LTRs are quite distinct from those of the *A. niger* CBS 513.88 ANiTA1 retrotransposon (cf. [51]). We have given the new *A. niger* Ty1/copia transposon in CBS 147324 the name ANiTA2. In strain CBS 133816, a solo LTR—100% identical to those of the autonomous ANiTA2 copy in CBS 147324—is observed at the same integration site between the (same) hexamer TSDs 5′-AGAAAC, suggesting that the retrotransposon is eliminated from the CBS 133816 genome by recombination.

The third mutant *aoxB* allele is the result of a point mutation that changes the start codon into CUG (Met1Leu) (Figure 3: Group IV). We found this missense mutation in nine strains (September 2022). If a protein can be produced using the next-in-frame AUG in the *aoxB* transcript (either Met18 or Met23) as its start codon, the mitochondrial target peptide is truncated by at least 17 AAs, i.e., one-third of the predicted mitochondrial target peptide would be lost, including a trimer of positively charged amino acids {RKR}, and, consequently, it may no longer function properly as a mitochondrial target sequence.

The fourth mutant *aoxB* allele we identified is due to a single-base deletion of a T in the DNA at the wobble position of the Val328 codon near the 3′ end of exon 3, at ~95% of the coding sequences (Figure 3: Groups V and VI). Consequently, a frameshift occurs after Pro329, yielding a 345 AA long protein with a completely different C-terminus (16 AA). We found four strains with this frameshift mutation, all of them sequenced recently [29]. Two of these strains, CBS 131.52 and CBS 769.97, also carry a nonsense mutation at the other end of the coding region in exon 1, resulting in a premature ochre stop codon at the position of Ser38 at ~11% of the gene product, within the mitochondrial target signal. These two latter strains thus harbour the sixth and only double-mutant allele of *aoxB*.

### 3.6. aoxB Pseudogenes Occur in (Most) Aspergillus tubingensis but Not in Other Named Members of the Aspergillus luchuensis Clade

The acquisition of the four-membered gene cluster with the divergent *aoxB*–*andB* genes in its centre is likely to have taken place earlier then the speciation events separating the *Aspergillus niger* clade in the series Nigri from the *Aspergillus luchuensis* clade [28,52]. A TBLASTN screen for the presence of the ensemble of the four genes (NB. Queries were the four gene products from strain ATCC 1015) resulted in the identification of a fraction of the *A. luchuensis* clade in which a remnant of the gene cluster has been retained in most isolates named *Aspergillus tubingensis*, including the strain in WU-2223L ([53]: WGS Master Accession BLWE). In addition, this clade also contains multiple isolates of “*Aspergillus niger sensu lato*”, such as strain An76 ([54], WGS: BCMY) and strain 3.316 (WGS: JAALJE). Within the identified group of isolates of black aspergilli, the two outward genes of the four-membered cluster (cf. Figure 2a), encoding for a conserved zinc-cluster transcriptional regulator and a protein with an N-terminal 3-hydroxyacyl-CoA dehydrogenase domain, respectively, are intact in the genomes listed in Table 2 (*sensu lato* isolates, separate column at the right of the table). However, the *andB* gene is severely degenerated in all listed genomes with numerous frameshifts, and a sizeable deletion is apparent between introns 2 and 4. The *aoxB* pseudogene features two or three frameshifts in exons 2 and 3, respectively. Nevertheless, the similarity between the full-length *aoxB* gene (*A. awamori*) and the pseudogene apparent in a fraction of isolates in the *A. luchuensis* clade (Supplementary Figure S6) remains high at ~85% (introns included).

Other named genome-sequenced species in the *A. luchuensis* clade *(A. luchuensis*, *A. piperis*, *A. vadensis*, *A. eucalypticola* and *A. neoniger*) and at the basis of the series Nigri (*A. brasiliensis*) do not have orthologues of any of the four genes of the plausible cluster (not shown), suggesting a prominent separation within the *A. luchuensis* clade based on the presence or absence of these four neighbouring genes, including *aoxB*.

## 4. Conclusions

The *Aspergillus niger aoxB* gene encoding a second alternative oxidase is divergently orientated from the *andB* gene encoding a paralogue of a type-II NADH dehydrogenase, constituting the *aoxB*–*andB* gene couple. These two genes are likely to be involved in parallel pathways to uncouple ATP synthesis by means of oxidative phosphorylation from NADH reoxidation, coordinated expression of which could in extremis allow control of metabolic reducing power without any concomitant build-up of proton motive force. A plausible distal origin of the *aoxB* paralogue outside the Aspergillaceae family coincides with a sporadic, scattered occurrence (i.e., in four species, three Aspergilli of different sections and one Penicillium) of the gene couple within the Aspergillaceae. This situation is congruent with the implication of one or more horizontal transfer events in the cluster’s dispersal as a credible alternative to vertical inheritance with myriad occasions of independent cluster loss in many diverged clades, even though the actual sources of the transferred DNA remain obscure. Different mutations in one gene, the *aoxB* gene, enable the categorization of the formal species *A. niger* (*sensu stricto*) into six groups of variants or cryptic species. Section Nigri rank third as identifiable causants of pulmonary aspergillosis, closely behind species of the section Flavi [6,8,9,10,55]. However, in some clinical studies causal *Aspergillus* spp. were not identified further. The existence of different *aoxB* alleles may contribute to a deeper resolution of the *A. niger sensu stricto* complex by molecular taxonomy in clinical settings, for example in cases of COVID-19-associated pulmonary aspergillosis [55].

## Figures and Tables

**Table 1:** *Aspergillus niger* strains used to certify the existence and sequence of *aoxB* alleles and the respective GenBank accession numbers.

*Aspergillus niger* Strain	Genome Master Accession	Source	Gene Allele	GenBank Accession Number(s)
ATCC 1015	ACJE01	Our laboratory	Wild type *aoxB* (Group I)	OQ606865 OQ686795
ATCC 1015	ACJE01	Our laboratory	Wild type *andB* *	OQ565583 * OQ689783 *
CBS 554.65	JAGRPH01	Matthias Steiger	Deletion part of *aoxB* and *andB* (Group II)	OQ590013
CBS 147343	JAKJMC01	Arthur Ram	Transposon insertion (Group III)	OQ633014
CBS 630.78	JAKJLX01	Arthur Ram	Frameshift mutation (Group V)	OQ633015
CBS 769.97	JAKJMK01	Arthur Ram	Frameshift mutation plus nonsense mutation (Group VI)	OQ633016

* The Group II deletion also affects the neighbouring *andB* gene. Therefore, we determined the sequences of the *andB* coding region and cDNA in the chosen “wild type” reference strain, ATCC 1015.

**Table 2:** Categorization of *Aspergillus niger sensu stricto* strains in accordance with six different alleles of the *aoxB* gene.

Group I	Group II	Group III	Group IV	Group V	Group VI	
Wild Type Allele aoxB	Allele 2 aoxB [Partial Deletion]	Allele 3 aoxB [Transposon Insertion]	Allele 4 aoxB [Missense Mutation Met1Leu]	Allele 5 aoxB [Frameshift near 3′ of CDS]	Allele 6 aoxB [Frameshift Plus Nonsense Mutation]	aoxB Pseudogene in *A. tubingensis* [*A. luchuensis* Clade]
IFM 58123 *A. awamori*	ATCC 13496	CBS 113.50	ATCC 13157 *A. phoenicis*	CBS 630.78	CBS 131.52	“*A. niger*” strain An76
ATCC 1015	CBS 101883 *A. lacticoffeatus*	CBS 118.52	JSC-093350089	CBS 147347	CBS 769.97	“*A. niger*” strain 3.316
ATCC 64974 [N402]	CBS 112.32	CBS 124.48	strain F1702			*A. tubingensis* WU-2223L
CBS 147345	CBS 115988	CBS 147322	strain M3604			*A. costaricensis* FKII-L6-BK-DRAB1
CBS 147346	CBS 115989	CBS 147323	strain R1650			*A. tubingensis* S/N-304-OC-P1
CBS 147482	CBS 147321	CBS 147324	strain R20-06			*A. tubingensis* S/N-308-IC-B1
CBS_139.54b *A. welwitschiae*	CBS 513.88	CBS 147343	strain RG13B1			*A. tubingensis* VS III B KN t
CCMB 663 *A. welwitschiae*	CBS 554.65 *A. niger* neotype	CBS 147344	strain S1603			*A. tubingensis* S/N-302-OC-P2
CCMB 674 *A. welwitschiae*	strain A1	CBS 147371	strain Y1650			*A. tubingensis* strain JS3-R1
DSM 1957	strain H915-1	strain F3_1F3_F				*A. tubingensis* strain JS3-P2
FDAARGOS_311	strain L2	ATCC 10864 (2 contigs) #				*A. tubingensis* strain C2-2
FGSC A1279	strain LDM3	CBS 133816 (2 contigs) #				strain Y4002A
IHEM 2864 *A. welwitschiae*	strain SH-2	CBS 147320 (2 contigs) #				strain S3103
ITEM 11945 (*A. welwitschiae*)		CBS 147352 (2 contigs) #				strain BSC-1
NRRL 3 [CBS 120.49; N400]		CBS 147353 (2 contigs) #				strain S1133
“*Penicillium fimorum*” S/N-308-OC-P1 *		strain MOD1-FUNGI2 (2 contigs) #				strain F8013-2
strain F3_4F1_F						strain S1118
strain F3_4F2_F						strain P1003-2
strain L14						strain PG3607
strain P1402						strain B7004A
strain S1						
strain Y2001A1						

Genomes of grey-marked clinical isolates were made public recently at NCBI. Their sequences are not used in *aoxB* phylogenies. * Genome sequences contaminated with *Aspergillus* DNA; two different *aoxA* genes present. **#** 5′- and 3′ parts separated by the transposon are split over two sequence contigs.

## Data Availability

Data are contained within the article and the associated Supplementary Materials. The determined *aoxB* and *andB* sequences were deposited at GenBank under accession numbers OQ565583, OQ590013, OQ606865, OQ633014–6, OQ686795 and OQ689783.

## Supplementary Materials

The following supporting information can be downloaded at: https://www.mdpi.com/article/10.3390/jof9050570/s1, Figure S1: Schematic overview of branched respiratory electron-transport chain in the mitochondrial inner membrane in filamentous fungi to illustrate the metabolic function of alternative oxidase (Aox) and type-II NADH dehydrogenase (And); Figure S2: The intron–exon structures of the two paralogous genes for alternative oxidase and for alternative NADH dehydrogenase (oxidizing cytosolic NADH) extant in *Aspergillus niger* strain ATCC 1015; Figure S3: The taxonomically aberrant position of the Onygenales AoxA clade is not a consequence of joint analysis of (orthologous) AoxA proteins with AoxB paralogues; Figure S4: Alignment of opposite LTRs of the non-autonomous and autonomous copies of ANiTA2 found amongst sixteen *A. niger sensu stricto* isolates with *aoxB* disrupted by retrotransposon insertion; Figure S5: Ty1/copia retrotransposon ANiTA2, autonomous copy 5129 nt; Figure S6: DNA similarity between the coding region of *A. awamori aoxB* and the *aoxB* pseudogene in *Aspergillus niger* (*sensu lato*) strain An76; Table S1: The intron–exon structure of *aoxB* and divergently orientated *andB* genes; Table S2: NCBI-lodged RNA sequence reads that cover exon–exon fusions in *aoxB* and *andB* in *A. calidoustus*, *A. implicatus* and *A. niger* strains (with full-length *aoxB* and *andB* genes); Table S3: Oligonucleotide primers used for PCR amplification of the *aoxB* and *andB* genes and cDNA prior to sequence verification of mutant alleles.
