# Supplementary material for: Mutations in the Second Alternative Oxidase Gene: A New Approach to Group Aspergillus niger Strains
Source: J Fungi (Basel). 2023 May 13;9(5):570. doi: 10.3390/jof9050570 (PMC10219456; doi:10.3390/jof9050570)
Supplement: Supplementary file 1 [file jof-09-00570-s001.zip › jof-2350013-supplementary.pdf]

Supplementary Material (online)

Mutations in the Second Alternative Oxidase Gene: A New Approach to Group *Aspergillus niger* Strains

Michel Flipphi [1,†], Alexandra Márton [1,2,†], Vivien Bíró [1,2], Norbert Ág [1],

Erzsébet Sándor [3], Erzsébet Fekete [1,*] and Levente Karaffa [1]

1 Department of Biochemical Engineering, Faculty of Science and Technology, University of Debrecen, H-4032 Debrecen, Hungary

2 Juhász-Nagy Pál Doctoral School of Biology and Environmental Sciences, University of Debrecen, H-4032 Debrecen, Hungary

3 Institute of Food Science, Faculty of Agricultural and Food Science and Environmental Management, University of Debrecen, H-4032 Debrecen, Hungary

* Correspondence: kicsizsoka@yahoo.com

†These authors contributed equally to this work.

Figure S1: Schematic overview of branched respiratory electron transport chain in the mitochondrial inner membrane in filamentous fungi to illustrate the metabolic function of alternative oxidase (Aox) and type-II NADH dehydrogenase (And).
Figure S2: The intron-exon structures of the two paralogous genes for alternative oxidase and for alternative NADH dehydrogenase (oxidizing cytosolic NADH) extant in *Aspergillus niger* strain ATCC 1015.
Figure S3: The taxonomically aberrant position of the Onygenales AoxA clade is not a consequence of joint analysis of (orthologous) AoxA proteins with AoxB paralogs.
Figure S4: Alignment of opposite LTR's of the non-autonomous and autonomous copies of ANiTA2 found amongst sixteen *A. niger sensu stricto* isolates with *aoxB* disrupted by retrotransposon insertion.
Figure S5: Ty1/copia retrotransposon ANiTA2, autonomous copy 5129 nt.
Figure S6: DNA similarity between the coding region of *A. awamori aoxB* and the *aoxB* pseudo gene in *Aspergillus niger* (*sensu lato*) strain An76.
Table S1: The intron-exon structure of *aoxB* and divergently orientated *andB* genes.
Table S2: NCBI-lodged RNA sequence reads that cover exon-exon fusions in *aoxB* and *andB* in *A. calidoustus*, *A. implicatus* and *A. niger* strains (with full-length *aoxB* and *andB* genes).
Table S3: Oligonucleotide primers used for PCR amplification of the *aoxB* and *andB* genes and cDNA prior to sequence verification of mutant alleles.

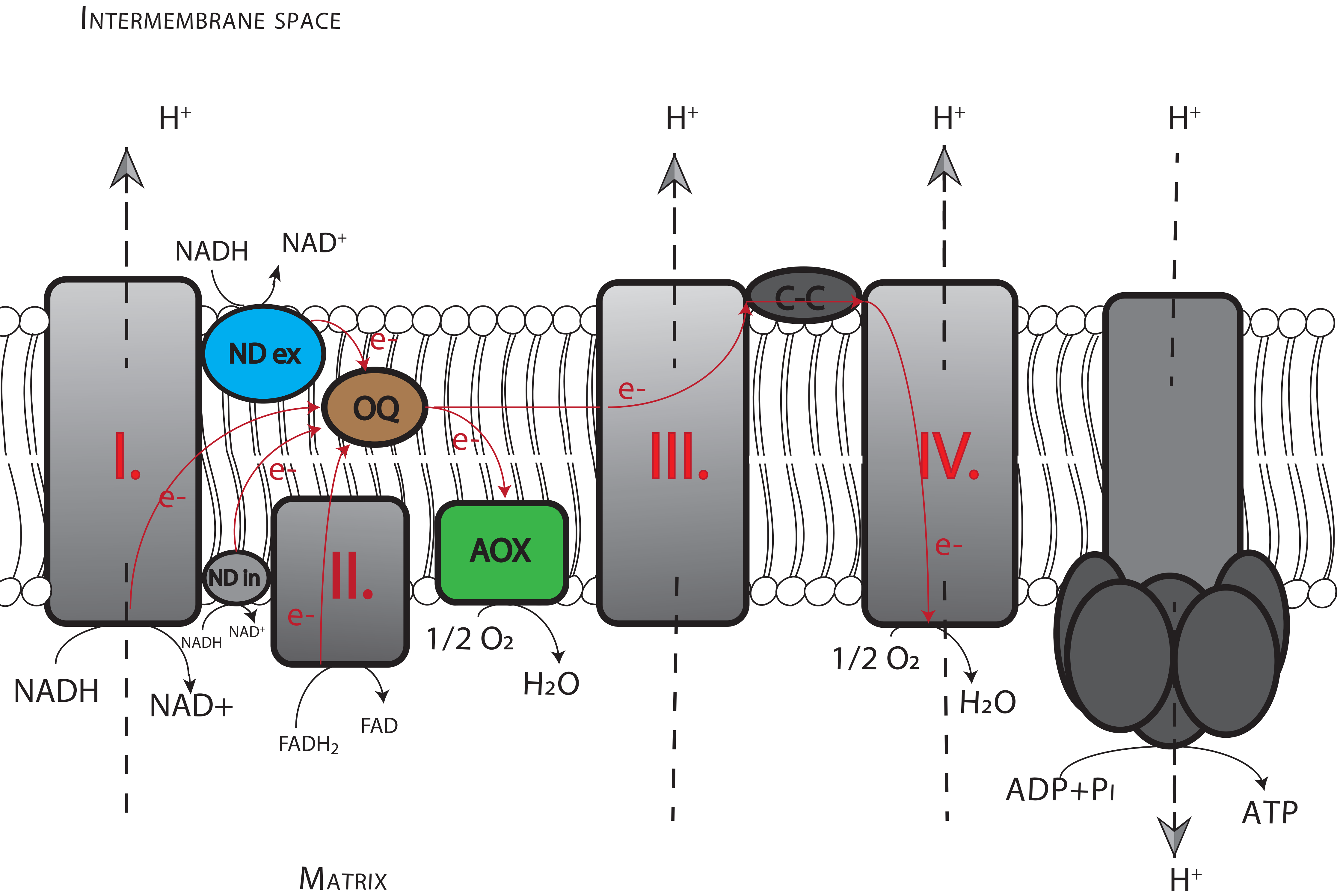

Figure S1. Schematic overview of branched respiratory electron transport chain in the mitochondrial inner membrane in filamentous fungi to illustrate the metabolic function of alternative oxidase (Aox) and type-II NADH dehydrogenase (And). The alternative electron fluxes are indicated in red. Associated translocation of protons over the inner membrane are shown. In the canonical chain, Type-I NADH:ubiquinone oxidoreductase (complex I), Ubiquinol:cytochrome C oxidoreductase (complex III) and Cytochrome C oxidase (complex IV), respectively, couple electron transport down the chain to proton translocation and the buildup of proton motive force that can be used to generate ATP by oxidative phosphorylation (at the right of the scheme). Complex II (succinate dehydrogenase) re-oxidizes mitochondrial FADH2 formed by the prior conversion of succinate to fumarate in the tricarboxylic acid cycle without pumping protons. Alternative oxidase (Aox; green) accepts electrons directly from the intermediate carrier ubiquinol (brown) and transfers them straight to molecular oxygen, bypassing the electrogenic, proton-translocating Complexes III and IV. NADH/NAD+ cannot pass the mitochondrial inner membrane. Alternative NADH dehydrogenases (And) or Type-II NADH:ubiquinone oxidoreductases oxidize either cytosolic (blue) or mitochondrial NADH (grey) and transfer the high-energy electrons to the ubiquinone intermediate (brown) without concomitantly pumping protons. Type-II alternative NADH dehydrogenases thus bypass the proton-translocating (Type-I) Complex I

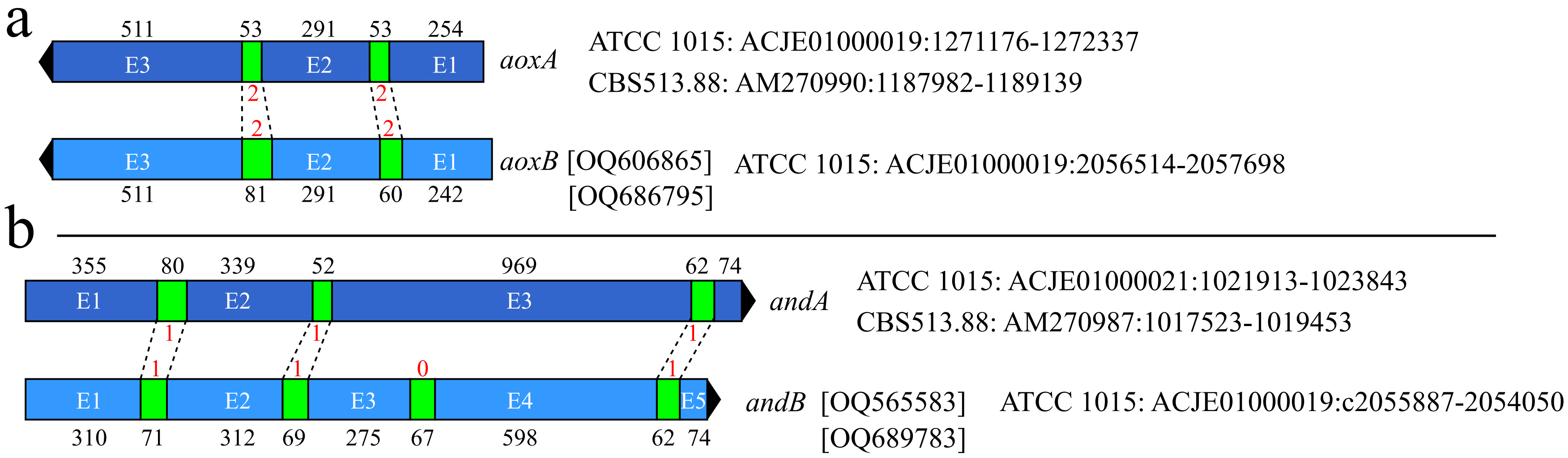

Figure S2. The intron-exon structures of the two paralogous genes for alternative oxidase and for alternative NADH dehydrogenase (oxidizing cytosolic NADH) extant in *Aspergillus niger* strain ATCC 1015. (**a**) Alternative oxidase genes, *aoxA* and *aoxB*. (**b**) Alternative NADH dehydrogenase genes, *andA* and *andB*. The exact location of the four genes in *A. niger* strain ATCC 1015 (sequence contig and coordinates) is mentioned to the right of the gene models. Relevant GenBank accession numbers are included. For the two ubiquitous "A" genes, the exact localisation in *A. niger* strain CBS 513.88 is also given. The exons of the coding regions (i.e., between ATG and STOP) are colored darker blue for the ubiquitous paralogous genes "A" and lighter blue for the rare paralogous genes "B". Introns are indicated by the green color and vertically connected with the dashed lines to emphasize intron position conservation. The arrowhead gives the orientation of the genes. The intron phase is indicated by the red digit (0; 1; 2). The phase-zero third intron is lost from the ubiquitous gene for the alternative NADH dehydrogenase, *andA*.

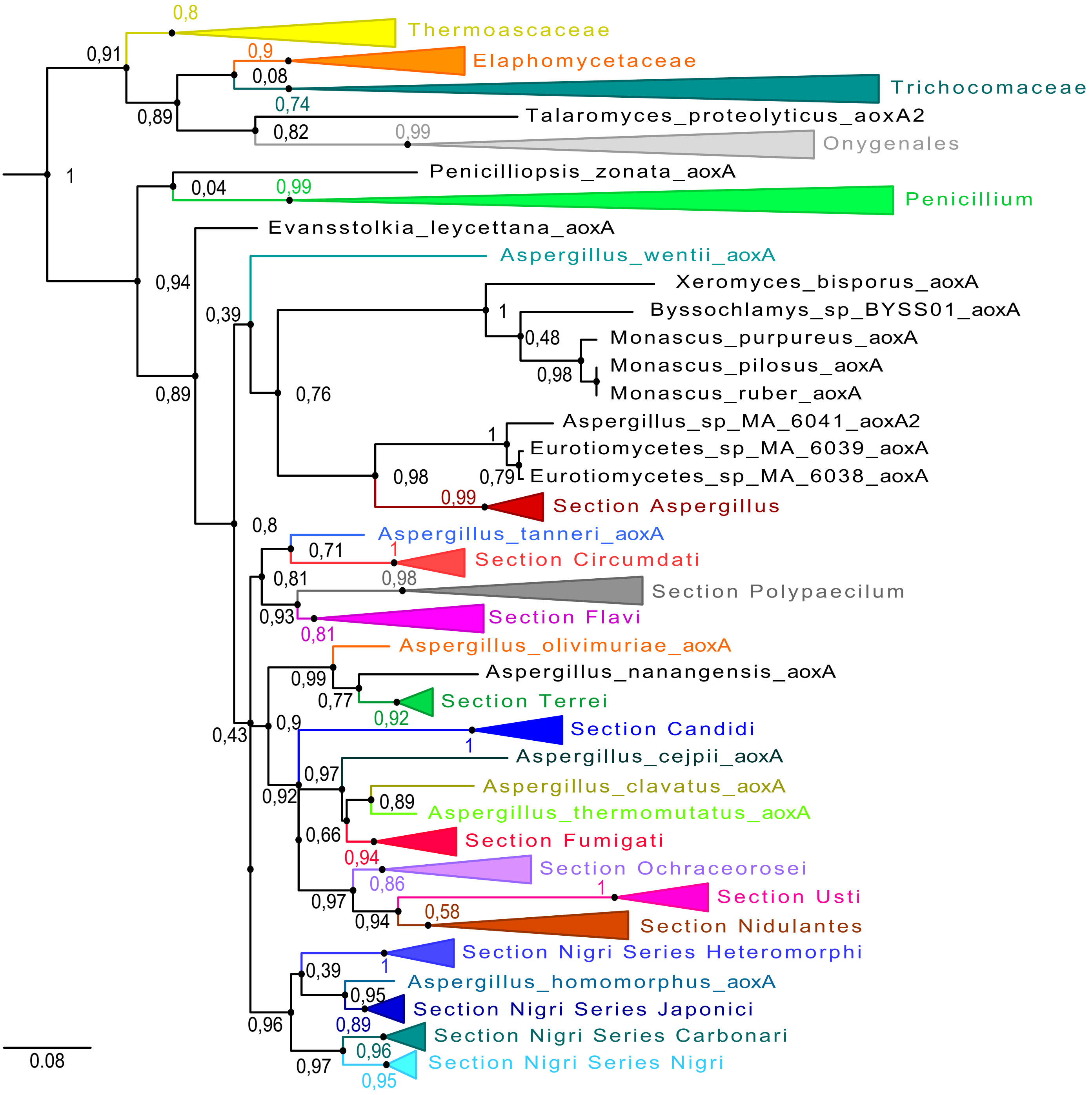

Supplementary Figure S3. The taxonomically abberant position of the Onygenales AoxA clade in maximum likelihood estimations of interrelations of Aox paralogs is not a consequence of joint analysis of (orthologous) AoxA proteins with AoxB paralogs. Essentially the same clade clustering was evident from control phylogenies of exclusively the AoxA imput (i.e., with the AoxB paralogs omitted). The Figure shows such a “AoxA-only”control tree constructed using exactly the same methodology for multiple sequence alignment (MAFFT), its trimming (BMGE) and the subsequent maximum likelihood inferrence (PhyML) (cf. legend to Figure 4b). The rooting outgroup of Lecanoromycetes AoxA was eliminated from view by subtree selection in FigTree. The clades specific to Thermoascaceae, Elaphomycetaceae, Trichocomaceae, Onygenales, the *Penicillium* genus, as well as the various *Aspergillus* sections were collapsed for a more convenient overview. However, section Nigri species were collapsed at the series level. Note the considerable evolutionary distance between the section Nigri series Nigri AoxA clade (at the bottom of the tree), the section Usti (includes *A. calidoustus*) and the estimated origin of paralogous AoxB as a sister clade of the Onygenales AoxA, near the top of the tree.

Supplementary Figure S4. Alignment of opposite LTR’s of the non-autonomous and autonomous copies of ANiTA2 found amongst sixteen A. niger sensu stricto isolates with aoxB disrupted by retrotransposon insertion (aoxB allele III)

```
-GAGAATGTTGAGGTAGTAACCCTGTAGGCTCAGTCACGTGGCTGCGCTCAGGCCGTCCACCAAGCGTCTCTTCGCGCTGGTTGCGCTGTGTGTTTCTAGATAGTAATATAATATACTTTTATTATATTCTTTACTTTTATTTCTTCCGTTTATAGTATTTAAACAGA-----
------| ||||| ||||||  || ||| || | | |  |  |  |||||| ||| |||||||||  ||||| || || ||||| ||| | ||| ||||||||||||| || | ||||||| |  |||||| ||||  |||||||||  | |||  ||  ||||||-----
------TATTGAGATAGTAATTCTATAGACTTAATTATATAATTATGCTCAGACCGCCCACCAAGCACCTCTTTGCACTAGTTGCACTGCGCGTTCCTAGATAGTAATACAACACACTTTTACTGCATTCTTCGCTTTCGTTTCTTCCGCCTGTAGTATTTAAACAGAGAATG
```

[118/162] 72.84 % ID **[copy disrupting aoxB]**

```
ATTTAATGTTGAGGCAGTAACCCTGTAGGCTCAGTCATGTGGCTGCGCTCAGGCCGCCCACCAAGCGCCTCTTCGCGCTGGTTGCGCTGCGTGTTTCTAGATAGTAATATAATATACTTTTACTATATTCTTCACTTTCGTTTCTTCCGCCTGTAGTATTTGAATAGA----
------|||||||  |||||  || ||  || | | || || ||| ||| || | ||| ||||||||||||||| ||||||||||||||| ||| |||||||||||||||| ||||||||| | |||||||  ||||||||||||||||||||||  || ||| |------
------TGTTGAGATAGTAATTCTATAAACTTAATTATATGACTGTGCTTAGACTGCCTACCAAGCGCCTCTTCGTGCTGGTTGCGCTGCGCGTTCCTAGATAGTAATATAACATACTTTTATTGTATTCTTTGCTTTCGTTTCTTCCGCCTGTAGCGTTCGAACATTTAAT
```

[131/162] 80.86 % ID

```
AGCTTTTGTTGAGGCAGTAACCCTGTAGGCTCAGTCACGTGGCTGTGCTCAGGCCGCCCACCAAGCGTCTCTTCGCGCTGGTTGCGCTGCGCGTTTCTAGATAGCAATACAATACACTTTTACTGTATTCTTTGCTTTTGTTTCTTCTGCTGGTAGCGTTTGAATAGATT----
------|||||||  ||||||| | ||| || ||||| || |||| |||||||| ||| |||||||| ||||||||||||||||| |||||||||||||||||||||||| ||||||||||| |||||| ||||| |||||||| || ||||| |||| ||| || ||----
------TGTTGAGATAGTAACCTTATAGACTTAGTCATGTAGCTGCGCTCAGGCTGCCTACCAAGCGCCTCTTCGCGCTGGTTGCACTGCGCGTTCCTAGATAGCAATACAACACACTTTTACTGCATTCTTCGCTTTCGTTTCTTCCGCCGGTAGCGTTCGAACAGCTTTTGT
```

[141/164] 85.98 % ID

```
CATTAGATGTTGAGGCAGTAACCCTGTAGGCTTGGTCACATGGCTGCGCTCAGGCCGCCCACCAAGCGCCTCTTCGCGCTGGTTGCGCTGTGCGTTTCTAGATAATAATACAACATACTTTTACTATATTCTTTACTTTCATTTCTTCTATTAATAATATTTAAATAGA-----
------||||| ||  |  |||  || ||  ||  || | ||||||  |||||||||||| || ||||||||||||||||||||||||||||||||||||||||| |||||||| |||||||||||  |||||| ||||| |||||||      ||   ||  || |-------
------ATGTTAAGATAATAATTCTATAAACTCAGTTATATGGCTATGCTCAGGCCGCCTACTAAGCGCCTCTTCGCGCTGGTTGCGCTGTGCGTTTCTAGATAGTAATACAATATACTTTTACTGCATTCTTCGCTTTCGTTTCTTCCGCCGGTAGCGTTCGAACATTAGATG
```

[125/163] 76.69 % ID

```
AGTTATTGTTGAGGAAGTAACCCTGTAGGCTCGGTCACGTGGCCGCGCTCAGACCGCCCACCAAGCACCTCTTCGCGCTGGTTGCACTGCGTGTTTCTAGATAGTAATATAACATACTTTCACTGCATTCTTTGCATCTGTTTCTTCTATTTATAGTTTGAATAGAT---
------|||| |   ||||| | | ||   |   | |  |  |   |||||| |||||||||||| |||||||    ||||||| |||   | || |||||||| |||| || | ||||||||||||||||||| ||||||||||| |  | ||||| ||| || |---
------TGTTAAAACAGTAATCTTATAAATTTAATTATATAACTATGCTCAGGCCGCCCACCAAGTACCTCTTTATACTGGTTGTGCTGTATATTCCTAGATAGCAATACAATACACTTTCACTGCATTCTTTGCGTCTGTTTCTTCCACCTGTAGTTCGAACAGTTATT
```

[116/161] 72.05 % ID

```
-ATCAGATGTTGAGGCAGTAATCCTGTAGGCTTGGTCACGTGGCTGCGCTCAGGCCGCCCACCAAGCGTTTCTTCGCGCTGGTTGCGCTGTGCATTTCTAGATAGTAATATAATATACTTTTACTACATTCTTCGCTTTCGTTTCTTCCGCCTGTAGTGTTTAAATAGA----
------|| || ||  ||||||  | |||  || || | ||||||| |||||||||||| || |||||  |||||| | |||||| |||| || ||||||||||||||||||||||||||||||| |||||||||||||||||||||||||||||||| |||   || |-----
------ATATTAAGATAGTAATTTTATAGATTTAGTTATGTGGCTGTGCTCAGGCCGCCTACTAAGCGCCTCTTCGTGTTGGTTGTGCTGCGCGTTTCTAGATAGTAATATAATATACTTTTACTGCATTCTTCGCTTTCGTTTCTTCCGCCTGTAGCGTTCGAACATCGAT-
```

[134/163] 82.21 % ID

```
-AAATCTGTTGAGGCAGTAACCCTGTAGGCTCGGTCACGTGGCCGCGCTCAGGCCGCCCACCAAGCGCCTCTTCGCGCTGGTTGCGCTGCGCGTTCCTAGATAGCAATACAACATACTTTTGCTGCATTCTTTGCGTTTATTTCTTCCTTTTGTAATTTAAATAAA-----
------|||| || |||||| ||| |||  |    |||| ||||| |||||||||||||||||||||||||||||||||||||||||||||||||||||||||||||||||||| |||||||||||||||||||||||||| |||||||||  |||| || ||| |||-----
------TGTTAAGACAGTAATCCTATAGATTTAATCACATGGCCACGCTCAGGCCGCCCACCAAGCGCCTCTTCGCGCTGGTTGCGCTGCGCGTTCCTAGATAGCAATACAATATACTTTTGCTGCATTCTTTGCGTTTGTTTCTTCCTCCTGTAGTTCAAACAAAATCT-
```

[142/160] 88.75 %

```
AGAAACTGTTGAGGCAGTAACCCTGTAGGCTCGGTCACGTGGCCGCGCTCAGGCCGCCCACCAAGCGCCTCTTCGCGCTGGTTGCGCTGCGCGTTCCTAGATAGCAATACAACACACTTTTGCTGCATTCTTTGCGTTTGTTTCTTCCTCCTGTAGTTCGAACAG-----
------|||||||||||||||||||||||||||||||||||||||||||||||||||||||||||||||||||||||||||||||||||||||||||||||||||||||||||||||||||||||||||||||||||||||||||||||||||||||||-----
------TGTTGAGGCAGTAACCCTGTAGGCTCGGTCACGTGGCCGCGCTCAGGCCGCCCACCAAGCGCCTCTTCGCGCTGGTTGCGCTGCGCGTTCCTAGATAGCAATACAACACACTTTTGCTGCATTCTTTGCGTTTGTTTCTTCCTCCTGTAGTTCGAACAGAAAC-
```

[159/159] 100 % ID **[autonomous copy ANiTA2: Ty1/Copia family]**

```
AGAAACTGTTGAGGCAGTAACCCTGTAGGCTCGGTCACGTGGCCGCGCTCAGGCCGCCCACCAAGCGCCTCTTCGCGCTGGTTGCGCTGCGCGTTCCTAGATAGCAATACAACACACTTTTGCTGCATTCTTTGCGTTTGTTTCTTCCTCCTGTAGTTCGAACAGAAAC-
```

Recombined LTRs (solo LTR with TSDs)
[autonomous copy recombined out: solo LTR 100 % identical]

The target site duplication (TSD) directly up- and downstream of the transposon LTR’s is highlighted by the green background.

Supplementary Figure S5. Ty1/copia retrotransposon ANiTA2, autonomous copy 5129 nt

AGACTGAGGCTGAGAGCGAGTGCAGACTTGGCTCGCTGCTGCAACGCGTGGCTCTTCGCAGCAGCTAACGATGCACATCCGTTGGCAAAAGATTGGCAAA**AGAAACTGTTGAGGCAGTAACCCTGTAGGCTCGGTCACGTGGCCG**
CGCTCAGGCCGCCCACCAAGCGCCTCTTCGCGCTGGTTGCGCTGCGCGTTCCTAGATAGCAATACAACACACTTTTGCTGCATTCTTTGCGTTTGTTTCTTCCTCCTGTAGTTCGAACAGgttatgagcccggcctctctgccgt
gaagctattcagactcgctagaacgcagtctttgaccccaaacgagacggagggcttacggccaccaaaggtggtgaacggagacgcgtcggtcttggcggttgcaatatccaataagaacacccttttgcgccaagggatacac
cattcgtcgatcctacccgtgctcaccactctaattggtcagcATGGCATTCGAAGTCGGACGCGGCGTTTCGTTTAAGAACCTTTCGCTTGAAGGGGAGGTGCCTACGGAGCCTACCACGCAGGCGGATAGGCTACCCGCGCAG
ATGCCTGATGGCGTTCCTGGCCTACCTCTCCTGCTACACGGACGCGAAAACTTCTCAAAATGGGACTTCCAGGTGAAGCAAGCACTTAAGAACGTCGGCTTGGAAGACCTGATCAAACTGGACCTGCCCAGACCTAGTGCCCAGC
ACGAAAAGTTCAAAGCATGGCACAGTTACTCCCGTGTTCTACAATCCTGGTTGACGGGCCAGCTATCGGGCAAGGTGATGGACCAGTTCCAGGCGTCCGCTGACAAAAAGGACTATGTCGACGATGCATATAAAGCAATACGAAG
AATCGTGATGGGCCACGGAATTGTCCTGTGCCAGAGCGTTGCTTACAACCTCGTTAAACCGAAGCGCTCGGACTATGCAACGGCGGGTGATTACATACAGGATTTCTGGAATAACTATGTCCTAGCCAAGGAGTTGAGGTGTGGG
ATACCCCCCTTTACAGCCTCGCTACTGCTCCTCCGAGAGATCCAAACGGATATGCCTGCCTGGGCCACCAACATGGAATGCAAGATGCCGGAGAATGCAGAGTCACTCTGTACAGATAATGACTTCTTTACTCTTTGCAGAAACG
CTATTGAACAGAGTAACAGAATAAGCGATATGGGATTGGCCGCCACTACCAAAAGCGGAAACCGGATCTCGTCATACAAATCAGATCCTGCAGGGGATGACTGGAAGAAGGCAACCTACCCGAAAAGAGGTGTTTCCAATGAAGA
CCACGCTGCAAAGATGCGAAATATAAAGCCGCAGTATGTTGACGGAGCTTGCGGTTACTGCAGATGCAAAGGTCATGGTCCTCCGAAGTGTTGGTATATTGAACCAACCATTCGTCCAACAGGCTGGGTGCCTTTTATAATGGAT
ATTTGGTTCTTCCGACCCAAAGGTGATCGTGGTAAGCGGAAGCAGGAACCAAAAGATGACTCTAAAGCGGTTGAGGCAGCACCTGCAAAGCAAATCGCTGAGATGTCTGCCCAACACGTTGCACTGCACGAAGCGGAGTCGGATG
ACGAAAATTTGCATATTTTAAATTTCTGTGGACTCGCCGCTGGGAACACCACTGAAGGGTCCCTGCGCTGGATCTACGATACTGGCAGTTCGCAGCACCTGACCCCTGACCGGGCCTCGCTAAACAACTACCACGACCTGGATGC
CTGCAACTTCTACAAGTATGGCACCTCTGATGGTGGCGTTGGTGTTGCAAAATCAGCAGGTTACCAGTCACTGTTCCTGTTCGCTAAAGAACGCCTTGAACTCAAGATTAAAGCTTACTACCAGCCGGGACTCGCCTACGGCCTC
CTTTCAGCGGACCGCCTGCGCAGGGATTTCGGTATATACGCCAACACGAAAGACCTCACCCTACGAAAGGAACAGGATGACAAAGTGGTGGGCCACCTGCAAGCATGCAAAAACGTGCTTTTCGTACGAACGGAAGATGCAGGGC
TGGCTCTGGCTGCAGTGGACCCCCTACTCCTGCACCGGCGCTACGGGCATGCAGGGTTCCGCAGAGTCACCTCCGCTGCGAAAAATCAACATATTGAACTCGAACACCGTGACGACCTCCACTGCGAAGCGTGTTCCCTAGGGAA
AGCGAAGCGGTTGGTATCCAGAGATCCGATCCCGAAGACCGCTGACCCTACCCAGCTGTGGCACGCTGACGTACAGCAAGTGACCCCAATCGGGCACGGAGGCTTCAAATACTTCCTGGTCATGGTCAATAACGCTACTCGTTTG
GTCCGTACGGCCATGCTGAAGCAGAAGAACCACGCCTCTACGGAGCTGATCCGAATGAATAACGAACACAAGAATATGGTTGGCCGCTACGTCGCCTCGTGGCACCTCGACGGGGGCCGTGAATTTAATAAATTCAAAGAGTGGG
CCAACCACGAAGATAGAGGAATTCGTGTTGTGATATCGCCTCCTAGAACACCTGAAGCCAACGGCCTCGCTGAGCGCTACGGCGGCTTGATCAACCAGATGAGCAGGGTGATGATGCTTGACTCGGGACTACCGGTGCACCTCTG
GCCGTTTGCGGTACGTCTGGCAGCGCAGATACTGAATCGGTTGGTTCCTACAGGAAGCGAAGTCAAACAAACGCCTGTTTCCCTATACAGATCATTCTACAACCTTCCGAATCCCGATGCCACCCTGGACTTCCTACGTGTGTTT
GGATGCCGCTGCTACCAACACATACCGGTAGAGGACCGCGTCACCTCCAGAAAGATGGGCCCCCGCGCCGATACTGGTTACTTTGTTGGCTTCGACGGGGACCACGGCCACGTGTATCTTGTCTGGTATCCTGCTTCGAATAAGG
TGAAGAAATCGCGTGACGTTACGTTCTATGAGGATCGCACGTATAAGGATGATCACGAAGATCCTGGTAGAACGCCTGCAGCGCCTACTCCTAAGGGTCCGATCATACCGGAGTCAGGCACCTCGATCAATTTTGATAACACAAT
TCTTCGTGCAATAAAGAATACAGAAATTGATAACGCTAAGGCCCTCCCTACACAGAATGACACCGCTGACGAACCCGTGCGCGGAAGAATACAGACTTTATCGAGTTCTACTGAACCCTCCGCACCTACCGGACCAATCCGCTCG
ATCGAGAATCCCGCCGATTACGAGGCTTCGTCTACCAAATACGCACATCGAGACTTCCGCCTTGCTGATTCGGAGCCCCCCTCTACTAAGGCCGTGGCTACCGCTCCAAGACGATCTCAACGTTCTACAAAAGGAAAGAGTCCTC
AGTGGCTAGGGAATGAACAAGTGGCTGCACTGGAGCAGGAATCCAACCTACCTCCCTTGCCTGCACTGAAACAACACGAAGTAGTCATACCGAACACCTATACGGAGGCCATTTCGTCACCGCTCGCTCCGTACTGGAAGGCGGC
CATGACCACACAGATCGATAAGATCAAATCAATGAAAACATATGATATCGTTGACCGGCCTGCTGGCGCTCGTGTGATCCCGGGCAAGTGGGTGTTTGACCTGAAAGTCAACACCGAAGGTGTTATTATACAATTTCGTGCTCGT
TGGGTTGTTTGTGGAAACCGCCAGGTCCCGGGCATCGATTACGACGAGTCTTTCGCCCCCGTGGCCAGCGAGATCGCCCTCAAAATGTTCATGACGAACGTGGCATTGAAACACCTCCGCTGGGAACAGTGTGATATCATTTCTG
CATACCTCCACGCTCAGATTGAAGGTAAACGTGTCTATATGAAGCAACCTACAGGTTATCACGAAGGGCCACCTGGGCGGGTCTGCCTGTTGAAAATGGCTCTCTACGGACTTCGCCAAGCGGCCCACCTCTGGAACCGCACCTT
AGACGAAAAGTTACAGGAGATTGGGTTCAAACCTCTGCCCGAAGACCCCTGCGTGTACAGAATGGGCGATATATGGCTGATAATCTATGTTGATGATTCCGCTATCGCAGGGCCAGAAGAGGAAGATCTAACCTCGGTCAAGGGT
CACCTTAACGAAGCGTTCGGCCTCAAGGAATTAGGCGAACCGAAGGTCCTGCTAGGGTGCAATGTTATACGTGATTACGAAGCAGGCACCATTTCACTATCCCAACCCACGTATGTGGAGGAGTGCCTAGCTGCCGCTAACCTAC
AAGCTTGCTCTGGCGTTGCTATTCCAATGACCCCTACCTACAAGAAGGCGGATCGTACAACAATGACGACTGATAATGTTGATATTAACGAATACCAGAGCCTCGTTGGTAGGCTGAATTGGTTGAGCACGAAATCACGCCCTGA
TCTCCGCCACGCTACCTTTCGCCTGCAGAGAAGGATGCACGATCCCACTACCGCTGACGTCAAAGCACTGAAGACCGTGTACAGATATCTCCGTGACACCGTTTCCTATGGGATCACTCTGGCCACCGATCATTCCAAAGGAATC
GAGGTCTACGTGGACGCCGCGCATGCGGATCACGAAGATGGCAAGTCCACTGAGGGTTACGTTATCTTCTACACCGGTGGCCCTATCACCTGGTCGTCGAACAAGCAAAGCGTTGTTGCGCCTTCCTCTACCGTTGTAGAATACT
GCGCATTTGATGCAGCCGTCAAGGACGTCTTATATGTCAAAAAGCTGGCACTGGCCTTTGACCTCAAGATATCGGATGACGGCAAGATACCCCTGTTCAGCGACGCCGCTAATTCGCTAAAGATATTGAACCATGGCGGCTACAC
CAAGTCGACTAGATGGCTCGATAATCGCTACCTATTCGTGGCCGATCTAATCAAAACGAACGCTATATCAATCCAACACGTTGCAGGTGTTTCCAACCCTGCTGACGGCCTCACTAAACCGCTGGATCGGGAAGCGTTCCGTACC
TTCAGGCGCCTGCTAGGAATGACCCCAGTCAGCGCCACCGATCTGCATCCTGGAAACTAGacttaatatgtgcaaaattgcctatttaggaaggaagtacggcttcaaataacacctccttgcacgcttcctcgcctagggggcc
g**TGTTGAGGCAGTAACCCTGTAGGCTCGGTCACGTGGCCGCGCTCAGGCCGCCCACCAAGCGCCTCTTCGCGCTGGTTGCGCTGCGCGTTCCTAGATAGCAATACAACACACTTTTGCTGCATTCTTTGCGTTTGTTTCTTCCTC**
CTGTAGTTCGAACAGAAACAAGTCATCGACAGGAAAGTGAGTGGCGGTGATGAACGAGACTTGATTGATTGGAACTGCCAGGGACGGGGTCCCATGACATCACCGCCGACCGGTCGGGA

LTR = 159 nt (purple: 5′ & 3′= 100 % identical); TSD = 6 nt 5′-AGAAAC (green).
ORF = 4512 nt (yellow); Integrase-RT-RNaseH polyprotein (Ty1/copia order) = 1503 AA

Supplementary Figure S6. DNA similarity between the coding regions of A. awamori aoxB and the aoxB pseudo gene in Aspergillus niger (sensu lato) strain An76

```
BDHI_Aawamori    ATGGTACGCCCAAATGCCATCCTTTTCAGCCCGATAAGCCGAAAGAGGTCAATGTACCTT   60
BCMY_An76        ATGATACGCCCGAATGCCGTCCTCTTCAGTCCGATAGGGCGAAGTAGGTCAATACATCCT   60

BDHI_Aawamori    ACAAACATGCATCGCATTTCATTGCACAACAGACCAGAAAAGCCCCTTAAATCAACACAG   120
BCMY_An76        CCAAACATGCATCGCATTTCATTGCGCAATAAACCAGGCAGACCACGGAAATTAACACAA   120

BDHI_Aawamori    CTCATACTTCTTGCTGGACAAGCGGGCTTTGCCACCTCAACTCCTTTATCATTCAAGTTG   180
BCMY_An76        CCGATACTTCTTGCTGGACAAGCGGGCTTTGCTACCTCCACTCCTTTATCATTCAAGCTG   180

BDHI_Aawamori    CAATCGCCACCAACAAAATTGACCCGAATCAAGGAGACAGCTATTGTCTGGAAACACCCA   240
BCMY_An76        CAATCACCACCAACAAAATCAACCCGAATCAAGAAGACGGCCATTGTCTGGAAACACCCA   240

BDHI_Aawamori    ATgtcagcccatatacctccctttcacttttttgtgtctgctctcctctaacactc----   295
BCMY_An76        ATatgagttcacctatctccctttcttttttggtgtctgctctttctctgctcttccctg   300

BDHI_Aawamori    --------ggaaaagCTATACAGAAAAGGAGATTCGAAATATCACTGTAGCTCATCGCGA   348
BCMY_An76        acaattaaaaaaacCTATACAGAGAAAGAGATTCCTGATATCACTGTGGCTCATGGCAA   360

BDHI_Aawamori    AGCCAAATGCCTTTCTGACAAGATCGCCTTGGCTTGGTACGTTTGCTTCGATGGTGCAC   408
BCMY_An76        TGCTAAGTGTCTTTCTGATAAGGTCGCCTTTGGCTTGGTACGTTGTCTTCGATGGTACAC   420

BDHI_Aawamori    TGATTTCGTGACTGGGTATCGAAATCCCGAAAGGAACAAAGAAATCGCAGACAAGTTTGA   468
BCMY_An76        TGATTTTGTGACTGGGTATCGAAATCCTGAAGGGAA-AAAGAAGTCGTAGACAAGTTTGA   479

BDHI_Aawamori    TATGACTGAGAGAAAATGGCTTATTCGTTTCATTTTTCTCGAATCGGTTGCTGGGGTGCC   528
BCMY_An76        GATGACTGAGACAAAGCCACTTATTAGTTTCACCATTCTCGAATCGGTTGCAG-GGTGCC   538

BDHI_Aawamori    TGGTATGGTGGGCGGCATGCTCCGACATCTGAAGAGTATCAGACGGATGAAGAGGGATCA   588
BCMY_An76        CGGTATGGTGGGCGGCATGCTCCGACATTTAAAGAGTGTTAGACAGATGAAGAAGGATCC   598

BDHI_Aawamori    TGGATGgtcagtacaagggaaaaaaa-t-aattatctccaagtacttagcagtagctaca   646
BCMY_An76        TGGATGgtcagtgcgagggaaaaaaaataaaatatctttcaaaatgttaaaagtagctaca   658

BDHI_Aawamori    caagaagctgcagactgacacaaccgcagGATCGAGTCCTTGATTGACGAAGCCTACAAC   706
BCMY_An76        caagaagctgctgactgacacaacttgcagGATTGAGTCCTTGATTGAAGAAGCCTACAAC   718

BDHI_Aawamori    GAGCGCATGCACCTCTTGACATTCCTCGAACTGGCAGATCCAGGTATCTTCATGCGCTTA   766
BCMY_An76        GAGCGCATGCACCTCTTGACATTCCTCGAACTGGCAGATCCAGGTATAATTCATGCGCTTC   778

BDHI_Aawamori    GTTGTCTTGGCCGCGCAGGGCCGTCTTCTTCAATGCCTTCTTCGTCTCCTACCTTGTTTCG   826
BCMY_An67        ATGGTCTTGGCCGCGCAGAGCCTCTTTTTTAATGCCTTCTTTGTCTCCTATCTCGTTCTA   838

BDHI_Aawamori    CCCAAGACATGTCACCGGTTTGTTGGATACCTAGAGGAAGAAGCTGTCATTACCTATACA   886
BCMY_An76        CCCAAAACATGTCACCGGTTCGTCGGATACCTACAGGAAGAAGCTGTCATTACCTTTACA   898

BDHI_Aawamori    CACGCAATCCGTCAACTTCAAGCTGGAAAACTGCCAGCATGGGACAATTTATCAGCACCG   946
BCMY_An76        CCTGCAATCCATGAACTCCAAGCTGGAAAGCTGCACGCATGGGACGATCTACCAGCACCG   958

BDHI_Aawamori    GAGATTGCAATCAAATATTGGAGAATGCCAGAAGGAAAACAGAGAATGGTCGATTTGTTG   1006
BCMY_An76        GAGATTGCAGTCAAAGATTGCAGAATGCCAAAGGGAAAACAGAAGATGCTTGATTTGTTG   1018

BDHI_Aawamori    CTTTATGTTCGGGCGGATGAAGCGAAGCATCGCGAGGTTAACCATACTTTGGGAAACTTA   1066
BCMY_An76        CTCTATGTTCGGATGGATGAAGCGAAGCATCGCGAGGTGAACCATACTTGGGGA-----   1073

BDHI_Aawamori    GACCAGCATGAAGATCCCAATCCTTATACTGCGCTTTATCACGATGGGAATAATGCTGGT   1126
BCMY_An76        ---CAGCAAGAGGATCCCAATCCTTATACTGCGCTTTATCACGAAGGGAATAATGTTGGT   1130

BDHI_Aawamori    CCCTCAGCGTCGCCCGATGCAGGGAAGCCTATTGGATGGGAGCGAGATGAACTAATTTAG   1186
BCMY_An76        TCCTCAGCGTCGCCTATCGCACGGAAGCCTTTTGGGTTAGAGCGAAATGAAGTAATTTAG   1190
```

Three frameshift mutations are indicated by the hyphen in the red background in the aligned An76 sequence as opposite to the green background in the A. awamori sequence. The 5'-donor, lariat branch point- and 3'-acceptor sequences in both introns are given in red lettering. These three sequence elements in the seemingly noncanonical first intron in An76 are in the turquoise background.

Supplementary Table S1: The intron-exon structure of aoxB and divergently orientated andB genes from the start to the stop codon. The sequence contig and the coordinates precisely localise (the coding parts of) the exons for each gene in each fungus. The phase and size of the position-conserved introns separating consecutive exons are given. For Aspergillus implicatus and Penicillium swiecicki, the data refer to the sequence assemblies accessible at the Mycocosm website of the Joint Genome Institute (US Department of Energy). We did not use the results from autoannotation: such predictions prove incorrect in Aspergillus niger ATCC 1015, NRRL3 and CBS 513.88 for both aoxB and andB.

		**exon1**	**intron1**	**exon2**	**intron2**	**exon3**	**intron3**	**exon4**	**intron4**	**exon5**
**A.niger<br>ATCC1015<br>andA**	**NA**	ACJE01000021<br>1021913-1022267<br>355 bp	phase 1<br>80 bp	ACJE01000021<br>1022348-1022686<br>339 bp	phase 1<br>52 bp	ACJE01000021<br>1022739-1023707<br>969 bp	phase 1<br>62 bp	ACJE01000021<br>1023770-1023843<br>74 bp		
**A.niger<br>ATCC1015<br>aoxA**		ACJE01000019<br>1271176-1271429<br>254 bp	phase 2<br>53 bp	ACJE01000019<br>1271483-1271773<br>291 bp	phase 2<br>53 bp	ACJE01000019<br>1271827-1272337<br>511 bp				
	**ATG-CAT***	**exon1**	**intron1**	**exon2**	**intron2**	**exon3**	**intron3**	**exon4**	**intron4**	**exon5**
**A.niger<br>ATCC1015<br>andB**	626bp	ACJE01000019<br>c2055887-2055578<br>310 bp	phase 1<br>71 bp	ACJE01000019<br>c2055506-2055195<br>312 bp	phase 1<br>69 bp	ACJE01000019<br>c2055125-2054851<br>275 bp	phase 0<br>67 bp	ACJE01000019<br>c2054783-2054186<br>598 bp	phase 1<br>62 bp	ACJE01000019<br>c2054123-2054050<br>74 bp
**A.niger<br>ATCC1015<br>aoxB**		ACJE01000019<br>2056514-2056755<br>242 bp	phase 2<br>60 bp	ACJE01000019<br>2056816-2057106<br>291 bp	phase 2<br>81 bp	ACJE01000019<br>2057188-2057698<br>511 bp				
**A.awamori<br>IFM 58123<br>andB**	621bp	BDHI01000007<br>c2238650-2238341<br>310 bp	phase 1<br>71 bp	BDHI01000007<br>c2238269-2237958<br>312 bp	phase 1<br>69 bp	BDHI01000007<br>c2237888-2237614<br>275 bp	phase 0<br>67 bp	BDHI01000007<br>c2237546-2236949<br>598 bp	phase 0<br>66 bp	BDHI01000007<br>c2236882-2236809<br>74 bp
**A.awamori<br>IFM 58123<br>aoxB**		BDHI01000007<br>2239272-2239513<br>242 bp	phase 2<br>61 bp	BDHI01000007<br>2239575-2239865<br>291 bp	phase 2<br>81 bp	BDHI01000007<br>2239947-2240457<br>511 bp				
**A.welwitschiae<br>CCMB 674<br>andB**	625bp	PUWS01000026<br>128688-128997<br>310 bp	phase 1<br>71 bp	PUWS01000026<br>129069-129380<br>312 bp	phase 1<br>69 bp	PUWS01000026<br>129450-129724<br>275 bp	phase 0<br>67 bp	PUWS01000026<br>129792-130389<br>598 bp	phase 1<br>62 bp	PUWS01000026<br>130452-130525<br>74 bp
**A.welwitschiae<br>CCMB 674<br>aoxB**		PUWS01000026<br>c128062-127821<br>242 bp	phase 2<br>59 bp	PUWS01000026<br>c127761-127471<br>291 bp	phase 2<br>81 bp	PUWS01000026<br>c127389-126879<br>511 bp				
**A.calidoustus<br>SF006504<br>andB**	551bp	CDMC01000004<br>c622233-621924<br>310 bp	phase 1<br>49 bp	CDMC01000004<br>c621874-621536<br>339 bp	phase 1<br>67 bp	CDMC01000004<br>c621468-621194<br>275 bp	phase 0<br>73 bp	CDMC01000004<br>c621120-620526<br>595 bp	phase 1<br>73 bp	CDMC01000004<br>c620452-620379<br>74 bp
**A.calidoustus<br>SF006504<br>aoxB**		CDMC01000004<br>622785-623020<br>236 bp	phase 2<br>52 bp	CDMC01000004<br>623072-623362<br>291 bp	phase 2<br>70 bp	CDMC01000004<br>623433-623943<br>511 bp				
**A.implicatus<br>CBS_484.95<br>aoxB**	544bp	Aspimp1<br>scaffold_11<br>206645-206877<br>(- strand)<br>233 bp	phase 2<br>49 bp	Aspimp1<br>scaffold_11<br>206305-206595<br>(- strand)<br>291 bp	phase 2<br>61 bp	Aspimp1<br>scaffold_11<br>205733-206243<br>(- strand)<br>511 bp				
**A.implicatus<br>CBS_484.95<br>andB**		Aspimp1<br>scaffold_11<br>207421-207718<br>298 bp	phase 1<br>74 bp	Aspimp1<br>scaffold_11<br>207793-208131<br>339 bp	phase 1<br>64 bp	Aspimp1<br>scaffold_11<br>208196-208473<br>278 bp	phase 0<br>58 bp	Aspimp1<br>scaffold_11<br>208532-209144<br>613 bp	phase 1<br>60 bp	Aspimp1<br>scaffold_11<br>209205-209278<br>74 bp
**A.implicatus<br>CBS_484.95<br>aoxB2<br>[paralog]**	**NA**	Aspimp1<br>scaffold_2<br>49588-49847<br>(- strand)<br>260 bp	phase 2<br>57 bp	Aspimp1 scaffold_2<br>49255-49530<br>(- strand)<br>276 bp	phase 2<br>64 bp	Aspimp1<br>scaffold_2<br>48680-49190<br>(- strand)<br>511 bp				
**P.swiecickii<br>182 6C1<br>aoxB**	613bp	Penswi1<br>scaffold_5<br>470967-471232<br>266 bp	phase 1<br>82 bp	Penswi1<br>scaffold_5<br>471315-471605<br>291 bp	phase 0<br>60 bp	Penswi1<br>scaffold_5<br>471666-472176<br>511 bp				
**P.swiecickii<br>182 6C1<br>andB**		Penswi1<br>scaffold_5<br>469712-470354<br>(- strand)<br>643 bp	phase 1<br>62 bp	Penswi1<br>scaffold_5<br>469372-469649<br>(- strand)<br>278 bp	phase 0<br>57 bp	Penswi1<br>scaffold_5<br>468684-469314<br>(- strand)<br>631 bp	phase 1<br>59 bp	Penswi1<br>scaffold_5<br>468551-468624<br>(- strand)<br>74 bp		

Footnote: ATG-CAT* : The column specifies the distance between the start codons of the divergent aoxB and andB genes. NA: Not applicable. The ubiquitous aoxA and andA genes (top of the Table) are never linked. The A. implicatus aoxB2 gene is not linked to a type-II NADH dehydrogenase gene.

Supplementary Table S2. NCBI-lodged RNA sequence reads that cover exon-exon fusions in aoxB and andB in A. calidoustus, A. implicatus and A.niger (with full-length *aoxB* and *andB* genes).

Species Gene Intron	Database (NCBI)	SRA reads	Species Gene Intron	Database (NCBI)	SRA reads	Species gene Intron	Database (NCBI)	SRA reads
	ERX631993	ERR677102.16649058.2		ERX631996	ERR677105.6854148.2			
**A.calidoustus andB intron 1**<br>TTAGGCACT	ERX631995	ERR677104.15682812.1<br>ERR677104.15682812.2		ERX631995	ERR677104.2332297.1	**A.implicatus andB intron 1**<br>CTGGGCACT	SRX5208515	not covered
	ERX631996	ERR677105.2162584.1<br>ERR677105.2162584.2		ERX631994	ERR677103.2510794.2<br>ERR677103.1656912.1<br>ERR677103.900396.2	**A.implicatus andB intron 2**<br>TTTGGAATC	SRX5208515	SRR8399217.21403927.2<br>SRR8399217.387016.1
	ERX631988	ERR677097.373254.2	**A.calidoustus aoxB intron 1**<br>CCAATCTAC	ERX631993	ERR677102.2082084.2	**A.implicatus andB intron 3**<br>CCCTCGATTTTA	SRX5208515	not covered
**A.calidoustus andB intron 2**<br>TTTGGGATT	ERX631990	ERR677099.18680577.2<br>ERR677099.18680577.1<br>ERR677099.2869718.1		ERX631992	ERR677101.10577410.1<br>ERR677101.8218975.1<br>ERR677101.8193533.1	**A.implicatus andB intron 4**<br>AGTGTCCAG	SRX5208515	SRR8399217.2264778.1<br>SRR8399217.25258407.1<br>SRR8399217.992531.1<br>SRR8399217.23243498.1
	ERX631993	ERR677102.14622834.2						
	ERX631991	ERR677100.9382643.1						
	ERX631996	ERR677105.17252672.2<br>ERR677105.17252672.1		ERX631996	ERR677105.2013743.2			
**A.calidoustus andB intron 3**<br>CCTTCTGTGCTT	ERX631989	ERR677098.13859357.2<br>ERR677098.2998495.2<br>ERR677098.2998495.1<br>ERR677098.22770266.2<br>ERR677098.13859357.1	**A.calidoustus aoxB intron 2**<br>GGATGGATT	ERX631995	ERR677104.16482873.2<br>ERR677104.16482873.1	**A.implicatus aoxB intron 1**<br>CCAATCTAT	SRX5208515	SRR8399217.6320403.2
	ERX631992	ERR677101.1542532.2<br>ERR677101.1542532.1		ERX631994	ERR677103.1971655.2			
**A.calidoustus andB intron 4**<br>AGTGCCCGG	ERX631994	ERR677103.6474623.1<br>ERR677103.6474623.2		ERX631993	ERR677102.23350232.2<br>ERR677102.10276109.2<br>ERR677102.4764038.2<br>ERR677102.4764038.1	**A.implicatus aoxB intron 2**<br>GGATGGATC	SRX5208515	SRR8399217.23319380.2<br>SRR8399217.8293776.1<br>SRR8399217.6320403.1
	ERX631993	ERR677102.17188126.1<br>ERR677102.17188126.2<br>ERR677102.12370809.2		ERX631992	ERR677101.7231996.1			
**A.niger aoxB intron 1**<br>CCAATCTAC	SRX9722265	SRR13293334.22709813.2	**A.niger andB intron 1**<br>AGTGCCTAA	SRX11975055	SRR15678655.579521.2	**A.niger andB intron 3**<br>TAGGACAGA	SRX12789281	SRR16587860.3272965.1
				SRX11975055	SRR15678655.579521.1			
**A.niger aoxB intron 2**<br>GGATGGATC	SRX11975789	SRR15679403.6264149.1	**A.niger andB intron 2**<br>TATCCCAAA	SRX12789281	SRR16587860.708123.1	**A.niger andB intron 4**<br>CCGAGAGCT	SRX18320010	SRR22348904.213785.1
				SRX15688375	SRR19638148.2825246.1			

Footnote: Per database, a maximum of five SRAs covering the exon-exon junction after excision of the indicated intron are given.

Strain gene Intron	Database (NCBI)	SRA reads
**A.implicatus aoxB2 intron1** CCTGTATAC	SRX5208515	SRR8399217.31224079.1<br>SRR8399217.30796192.2<br>SRR8399217.30796192.1<br>SRR8399217.29964246.2<br>SRR8399217.29933532.2<br>SRR8399217.29533741.1
**A.implicatus aoxB2 intron2** GGGTGGATC	SRX5208515	SRR8399217.31331017.2<br>SRR8399217.31167892.1<br>SRR8399217.30842627.2<br>SRR8399217.29836882.2<br>SRR8399217.29657382.2<br>SRR8399217.29442532.1

Table S3. Oligonucleotide primers used for PCR amplification of the *aoxB* and *andB* genes and cDNA prior to sequence verification of mutant alleles.

**Strain**	**Primer**	**Sequence (5'-)**
Aspergillus niger ATCC 1015 *aoxB* and cDNA	1015-aoxB-5prime-Fw1	CGTTCGATCTTCTCGCTAAG
	1015-aoxB-intern-Rv1	GTGTATAGGTAATGACAGCT
	1015-aoxB-intern-Fw2	GTGCACTGATTTCGTGACTG
	1015-aoxB-3prime-Rv2	CCATAAAGAATAACACCTGA
	1015-aoxB-5prime-cDNA-Fw1	ATCCCTGCCGAGGTGTCCAA
	1015-aoxB-5prime-cDNA2-Fw1	GAGACAGCTATTGTCTGGAA
	1015-aoxB-intern-cDNA-Rv1	TGACAGCTTCTTCCTCCAGG
Aspergillus niger ATCC 1015 *andB* gene and cDNA	1015-andB-3prime-Fw1	GCTCGGTACCGAGGAAAGT
	1015-andB-intern-Rv1	GAGCAGAATGACACTGAGCT
	1015-andB-intern-Fw2	GGAAACAGTCTCTGTACATC
	1015-andB-5prime-Rv2	GAACCAGGCCAGATATACAGC
	1015-andB-5prime-cDNA1-Rv2	AGTAGAGGCAGATCCGTCAA
	1015-andB-5prime-cDNA2-Rv2	CCAGGCCAGATATACAGCCTA
Aspergillus niger CBS 554.65 *aoxB* and *andB*	1015-andB-3prime-Fw1	GCTCGGTACCGAGGAAAGT
	1015-aoxB-intern-Rv1	GTGTATAGGTAATGACAGCT
	554-65_andB-special-Fw1	GCAAGAGATCCTTGGTGAGT
	554-65_andB-special-Fw2	AGTTGCACTGTATCTCGTTG
Aspergillus niger CBS 147343 *aoxB*	1015-aoxB-5prime-Fw1	CGTTCGATCTTCTCGCTAAG
	554-65_aoxB-intern-Rv1	GTGTATAGGTGATGACAGCT
	1015-aoxB-intern-Fw2	GTGCACTGATTTCGTGACTG
	124-48_transposon-Rv1	CTACAGGGTTACTACCTCAA
	124-48_transposon-Fw1	CCGCCTGTAGCGTTCGAACA
	1015-aoxB-3prime-Rv2	CCATAAAGAATAACACCTGA
Aspergillus niger CBS 630.78 *aoxB*	630-78-aoxB-5prime-Fw1	CGTTTGATCTTCTCGCTAAG
	1015-aoxB-intern-Rv1	GTGTATAGGTAATGACAGCT
	1015-aoxB-intern-Fw2	GTGCACTGATTTCGTGACTG
	630-78-aoxB-3prime-Rw2	CCATAAGAATAACACCTGAA
Aspergillus niger CBS 769.97 *aoxB*	630-78-aoxB-5prime-Fw1	CGTTTGATCTTCTCGCTAAG
	1015-aoxB-intern-Rv1	GTGTATAGGTAATGACAGCT
	1015-aoxB-intern-Fw2	GTGCACTGATTTCGTGACTG
	630-78-aoxB-3prime-Rw2	CCATAAGAATAACACCTGAA
